# Life-course restructuring of infectious mortality in China, 2004–2021: a national surveillance study

**DOI:** 10.3389/fpubh.2026.1881091

**Published:** 2026-07-21

**Authors:** Liu Binglian, Chen Aowen, Yang Yuting, Huang Changquan, Zhang Xingping

**Affiliations:** Sichuan University Affiliated Chengdu Second People's Hospital, Chengdu Second People's Hospital, West China School of Medicine, Sichuan University, Chengdu, Sichuan, China

**Keywords:** China, HIV/AIDS, infectious mortality, life course, respiratory infections, tuberculosis, viral hepatitis

## Abstract

**Background:**

While China has achieved significant milestones in controlling infectious diseases, how mortality patterns have shifted across different life stages remains poorly understood. This study aims to delineate the evolving burden of infectious disease mortality to inform age-specific public health strategies.

**Methods:**

We analyzed national death surveillance data from China spanning 2004 to 2021. “Combined infectious mortality” was defined as the aggregate of communicable and parasitic diseases plus respiratory infections. Focusing on four key categories—respiratory infections, tuberculosis, viral hepatitis, and HIV/AIDS—we calculated crude and age-standardized mortality rates (ASMR). Log-linear regression models estimated annual percentage changes (EAPCs). Primary trend inferences were restricted to 2013–2019 (stable post-expansion period); 2020–2021 was treated separately due to the absence of a COVID-19 code.

**Results:**

During 2013–2019, mortality declined substantially faster in younger than in older age groups: EAPC was −14.70% (95% CI –16.96 to −12.38) for children aged 0–4 years, but only −1.62% (95% CI –3.66 to 0.46) for adults aged 75 + (interaction *p* < 0.0001). In 2021, over half (55.04%) of combined infectious deaths occurred in adults aged 75+. Cause hierarchy varied by age: respiratory infections dominated at both extremes; HIV/AIDS ranked highest among young adults (20–39 years), though its advantage was narrow; viral hepatitis predominated in middle-aged adults (40–59 years). HIV/AIDS mortality rose among 60–74 year-olds (EAPC 5.90% per year), with higher burden in rural and western regions.

**Conclusion:**

China is undergoing a distinctive epidemiological transition: infectious deaths are increasingly concentrated in older age groups, driven primarily by divergent age-specific mortality trends rather than population ageing alone. Public health strategies must extend beyond maternal-child health frameworks to address growing vulnerabilities in older populations—particularly respiratory infections and HIV in ageing rural communities.

## Introduction

Although infectious mortality in China is often framed as a dwindling residual within the broader epidemiological transition, such a characterization oversimplifies a complex reality. The evolution of infectious disease services depends on distinguishing between a general retreat of pathogens and their strategic redistribution across different age cohorts. With respiratory infections remaining a leading cause of global mortality, and TB, hepatitis, and HIV persisting as national priorities, it is clear that the infectious threat is evolving rather than evaporating (1–5).

The granular nature of China’s integrated mortality surveillance system enables a direct interrogation of these demographic transitions, grounded in a comprehensive and consistent national repository (6). However, existing literature tends to be bifurcated: researchers have either focused on aggregate infectious mortality trends (7), or scrutinized specific diseases in siloed isolation. This fragmentation obscures the broader, interconnected shifts occurring across the entire infectious disease spectrum (8–12), Furthermore, current evidence (9, 13–16), tends to offer static snapshots of specific age-restricted cohorts—most older adults—rather than capturing the dynamic life-course restructuring that occurs within a unified mortality framework.

A comprehensive interpretation of infectious trends necessitates an integrated analytical framework. While absolute death counts reflect the sheer scale of the service burden, crude rates and age-standardized metrics provide essential context for population-level impact and cross-period benchmarking, respectively. This multidimensional approach is complemented by evaluating proportional mortality, ensuring that the significance of infections is weighed against the broader all-cause mortality landscape.

Applying this holistic lens to China’s 2004–2021 surveillance data, we scrutinized the “life-course restructuring” of infections, with a particular emphasis on tuberculosis, hepatitis, HIV, and respiratory diseases. We explicitly account for the COVID-19 anomaly in our analysis; the respiratory mortality series after 2019 is treated as a distinct phase, shaped by the potent indirect effects of non-pharmaceutical interventions (NPIs) and broader fluctuations in disease notification systems (17–20).

## Methods

### Data source and study design

Our analysis is based on annual mortality surveillance tabulations (2004–2021) curated by the Chinese Center for Disease Control and Prevention, which provide comprehensive data on annual death counts, monitored populations, and age-standardization weights. A critical juncture occurred in 2013 when the surveillance infrastructure expanded from 161 to 605 points—an expansion that secured national and provincial representativeness by covering approximately 24% of the population. Given this substantial recalibration of the data source, we prudently interpreted 2013 as a structural boundary in the surveillance architecture rather than an organic epidemiological shift.

As this study relied exclusively on publicly accessible, de-identified, and aggregated data, it precluded any possibility of record linkage or the reconstruction of individual-level trajectories. Consequently, according to national and institutional guidelines, this study was exempt from ethics committee approval and the requirement for informed consent.

### Endpoints, metrics, and statistical analysis

The primary endpoint of this study was combined infectious mortality, defined within the surveillance hierarchy as the aggregate of communicable diseases, parasitic diseases, and respiratory infections. Within the respiratory category (Category 1.2), we focused exclusively on infectious etiologies, omitting non-infectious respiratory causes. For our disease-specific analyses, we prioritized respiratory infections, tuberculosis, viral hepatitis, and HIV/AIDS. To ensure longitudinal consistency following the 2009 coding transition, we defined viral hepatitis as the sum of hepatitis B and C, validating this against older pre-2009 codes in sensitivity analyses. Similarly, tuberculosis was tracked via continuous pulmonary tuberculosis codes and cross-checked against earlier respiratory tuberculosis classifications. We excluded parasitic and tropical infections from the disease-specific decomposition because their national mortality impact remained negligible and contributed little to the overall restructuring of life-course mortality.

For each endpoint, we calculated deaths, crude mortality rates (CMRs), age-standardized mortality rates (ASMRs), and the percentage of all-cause deaths. We treated these measures as complementary lenses rather than interchangeable figures: while CMRs and death counts illustrate the real-world burden managed by populations and health services, ASMRs allow for comparisons that aren’t skewed by shifting age demographics. Furthermore, looking at the percentage of all-cause deaths helped us identify whether an infectious cause remained a significant threat within a specific life stage, even when absolute mortality rates were low. To capture these shifts across the lifespan, we stratified the population into six groups: 0–4, 5–19, 20–39, 40–59, 60–74, and 75 years or older.

We utilized log-linear regression to estimate annual percentage changes (EAPCs) for trend summaries. Although we present the full data series from 2004 to 2021 to provide descriptive historical context, we explicitly designate 2013 as the cutoff point for the surveillance system (expansion), rather than an epidemiological turning point. In 2013, the surveillance system underwent a significant expansion from 161 to 605 surveillance sites, resulting in substantial changes in the monitored population. Therefore, all primary trend analyses—including the main summary, the Results section, and all estimated annual percentage changes (EAPCs) presented in Table 1—are restricted to the relatively stable period of 2013–2019 after the system expansion. The full-period (2004–2021) EAPCs are presented only in the Supplementary Tables S5, S6 as a descriptive summary and are not used for primary inference. In addition, due to the absence of a specific disease code for COVID-19 and the potential changes in disease coding and healthcare-seeking behavior caused by the pandemic, data for 2020–2021 are presented separately and are not included in the trend estimates. We avoided deriving a bridging factor because the public aggregated data did not allow us to reconstruct pre-2013 rates to fit the post-2013 framework; therefore, EAPCs for the full 2004–2021 period serve as descriptive context rather than the basis for our main inferences (Table 2).

**Table 1:** National burden and temporal change for combined infectious mortality and the four major component causes, China, 2004–2021.

Disease	Deaths 2004	CMR 2004	ASMR 2004	Share 2004 (%)	Deaths 2021	CMR 2021	ASMR 2021	Share 2021 (%)	EAPC 2013–2019	2019 → 2020 change (%)
Combined infectious mortality	18,611	26.15	30.81	4.32	41,174	15.37	10.27	2.17	−0.37 (−0.93 to 0.19)	−20.08
Respiratory infections	8,733	12.27	14.86	2.03	24,026	8.97	5.46	1.26	0.71 (−0.43 to 1.86)	−27.51
Tuberculosis	4,314	6.06	7.05	1.00	4,628	1.73	1.25	0.24	−2.75 (−3.60 to −1.89)	−11.28
Viral hepatitis	2,955	4.15	4.81	0.69	6,873	2.57	1.91	0.36	−1.24 (−3.29 to 0.86)	−1.66
HIV/AIDS	158	0.22	0.23	0.04	1,406	0.52	0.48	0.07	3.65 (2.40 to 4.92)	−9.21

CMR = crude mortality rate (per 100,000 population). ASMR = age-standardized mortality rate (per 100,000 population). EAPC = estimated annual percentage change based on log-linear regression of annual CMRs, calculated for the stable post-expansion period 2013–2019. The 2004 and 2021 values are shown for descriptive comparison, but full-period (2004–2021) EAPCs are not presented here due to the 2013 surveillance system discontinuity; they are available in Supplementary Table S5. 2019 → 2020 change (%) = percentage change in CMR from 2019 to 2020; these values are presented as observed but are not incorporated into trend estimation due to the absence of a separate COVID-19 code and potential pandemic-related changes.

**Table 2:** HIV/AIDS mortality by broad age group at selected time points.

Age group	Year	Deaths	CMR (/100,000)	Share of all-cause deaths (%)	Share within combined infectious mortality (%)
0–4 years	2004	3	0.08	0.03	0.14
0–4 years	2013	7	0.06	0.04	0.31
0–4 years	2019	2	0.01	0.02	0.16
0–4 years	2021	1	0.01	0.01	0.18
5–19 years	2004	4	0.02	0.06	0.87
5–19 years	2013	14	0.04	0.13	2.40
5–19 years	2019	7	0.02	0.07	1.34
5–19 years	2021	10	0.02	0.11	3.48
20–39 years	2004	71	0.28	0.27	5.51
20–39 years	2013	282	0.41	0.56	14.93
20–39 years	2019	337	0.44	0.78	18.62
20–39 years	2021	303	0.44	0.77	22.49
40–59 years	2004	71	0.41	0.09	2.26
40–59 years	2013	465	0.63	0.19	6.49
40–59 years	2019	664	0.76	0.26	8.71
40–59 years	2021	618	0.74	0.24	9.40
60–74 years	2004	7	0.11	0.01	0.15
60–74 years	2013	183	0.71	0.04	1.88
60–74 years	2019	377	0.93	0.07	3.14
60–74 years	2021	323	0.79	0.06	3.32
75 + years	2004	2	0.11	0.00	0.03
75 + years	2013	78	0.87	0.01	0.34
75 + years	2019	124	1.03	0.01	0.41
75 + years	2021	151	1.07	0.01	0.67

CMR = crude mortality rate. Share within combined infectious mortality = percentage of combined infectious deaths attributable to HIV/AIDS within the same age group and year (denominator = all combined infectious deaths in that age group and year).

We calculated two versions of the estimated annual percentage change (EAPC). For assessing trends in age-specific mortality risk, we used age-standardized EAPC based on the 2010 Chinese census standard population. For describing trends in the overall population burden, we used EAPC based on crude rates. Unless otherwise specified, all EAPC estimates reported and interpreted in the main text are based on age-standardized rates.

To quantify the role of age structure in shaping the contemporary mortality burden, we conducted a cross-sectional analysis using 2021 data (Table 3), comparing crude mortality rates (CMR, reflecting actual age structure) with age-standardized rates (ASMR, removing age-structure effects). The age-structure contribution was calculated as (CMR − ASMR) / CMR × 100%, with positive values indicating an older-than-standard age structure that inflates observed mortality. This analysis was applied across regions, residence, and sex, and is unaffected by the 2013 surveillance expansion because it uses data from a single year.

**Table 3:** Age-structure contribution to combined infectious mortality, China, 2021.

Dimension	Group	CMR	ASMR	Age-structure contribution (%)
Region	Eastern	14.75	8.72	40.9
Region	Central	12.39	8.47	31.6
Region	Western	20.07	15.16	24.5
Residence	Urban	17.18	10.85	36.8
Residence	Rural	14.43	9.96	31.0
Sex	Male	18.91	14.18	25.0
Sex	Female	11.72	6.72	42.7

CMR = crude mortality rate (per 100,000 population); ASMR = age-standardized mortality rate (per 100,000 population). Age-structure contribution = (CMR − ASMR) / CMR × 100%.

As a supplementary historical-context analysis, we also applied the Kitagawa decomposition method (26) to data from 2004 and 2021 to explore the potential contributions of population ageing versus changes in age-specific mortality risk. However, because this comparison spans the 2013 surveillance expansion, it is presented in Supplementary Table S27 for historical context only and is not used for primary inference.

Finally, we conducted several sensitivity analyses to ensure our conclusions were not driven by external distortions or methodological choices. These included excluding the 2020–2021 pandemic years, comparing respiratory infections across upper and lower subsets, and assessing coding continuity for tuberculosis and viral hepatitis. We also examined HIV/AIDS patterns using more granular 5-year age bands. These steps allowed us to confirm that our primary findings remained robust, regardless of coding transitions, pandemic-related spikes in respiratory data, or the breadth of our age groupings. We also performed a sensitivity analysis using the WHO World Standard Population (2000–2025) to assess the robustness of our ASMR estimates to the choice of standard population (Supplementary Table S28).

### Ethical approval

This study used only public, de-identified, aggregated mortality data. No individual-level human or animal data were accessed. Ethics approval and informed consent were not required.

### Role of the funding source

**Figure 1:**
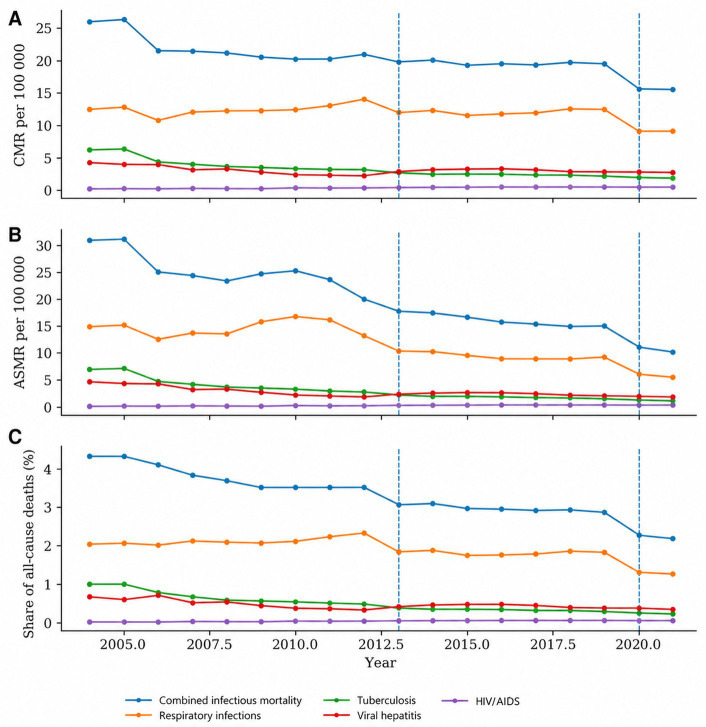
National trends in infectious mortality metrics, China, 2004–2021. **(A)** Crude mortality rates. **(B)** Age-standardized mortality rates. Dashed vertical lines indicate the 2013 surveillance system expansion (from 161 to 605 points) and the 2020 pandemic onset. Data before and after 2013 are not directly comparable in level; trend comparisons should focus on periods within each surveillance era. Primary trend analyses are restricted to 2013–2019.

The funder had no role in data acquisition, data analysis, data interpretation, writing of the report, or the decision to submit for publication (Figure 1).

## Results

### National and life-course patterns

Over the full 2004–2021 period, combined infectious mortality declined from 26.15 to 15.37 per 100,000, and ASMR fell from 30.81 to 10.27. However, due to the 2013 surveillance system expansion, these full-period changes should be interpreted with caution. We therefore base our primary trend inferences on the stable post-expansion period 2013–2019. During 2013–2019, the EAPC for combined infectious mortality was −0.37% (95% CI -0.93 to 0.19), indicating little contemporary change. Full-period (2004–2021) EAPCs are provided in the Supplementary Tables S5, S6 for descriptive context only.

**Figure 2:**
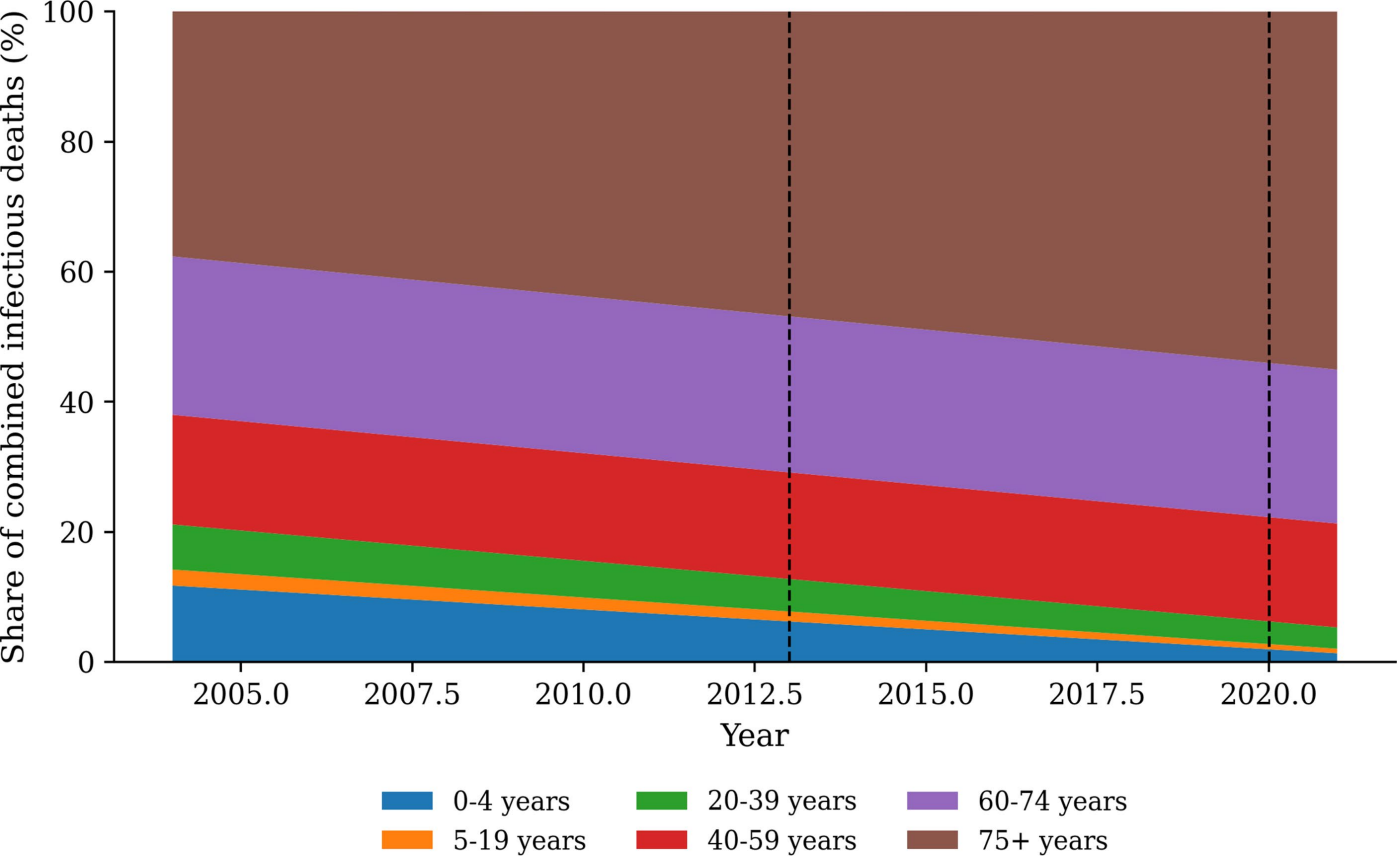
Age redistribution of combined infectious deaths, China, 2004–2021. Areas show the percentage contribution of each broad age group to total combined infectious deaths at the two time points (2004 and 2021) shown in the Supplementary Tables.

From 2004 to 2021, the age distribution of deaths involving infectious diseases shifted significantly toward older age groups (Figure 2). Among adults aged 75 years and older, the proportion of deaths involving infectious diseases increased from 37.67 to 55.04%, whereas among children aged 0–4 years, it dropped sharply from 11.75 to 1.35%. However, since the mortality surveillance system underwent a substantial expansion in 2013 (increasing from 161 to 605 surveillance sites), direct comparison of age-specific mortality ratios across the entire period from 2004 to 2021 is not appropriate. Therefore, we focused on age-specific trends during the stable period of the system after 2013.

Between 2013 and 2019, the estimated annual percentage change (EAPC) in infectious disease–related mortality among children aged 0–4 years was −14.70% (95% CI: −16.96 to −12.38), while that among adults aged 75 years and older was only −1.62% (95% CI: −3.66 to 0.46) (Supplementary Table S15). The decline in mortality was much steeper in the younger age group, and the interaction between age group and calendar year was highly significant (*p* < 0.0001; Supplementary Table S21). These findings indicate that, during the modern surveillance period, the mortality gap between older and younger populations has widened considerably, reflecting a true divergence in the risk of death from infectious diseases across the lifespan that goes beyond what can be explained by population aging alone. This age-based redistribution—rather than a synchronized decline across the lifespan—constitutes the defining characteristic of China’s infectious mortality transition. A supplementary historical decomposition spanning 2004–2021 is provided in Supplementary Table S27 for reference.

### Cause hierarchy across the life course

**Figure 3:**
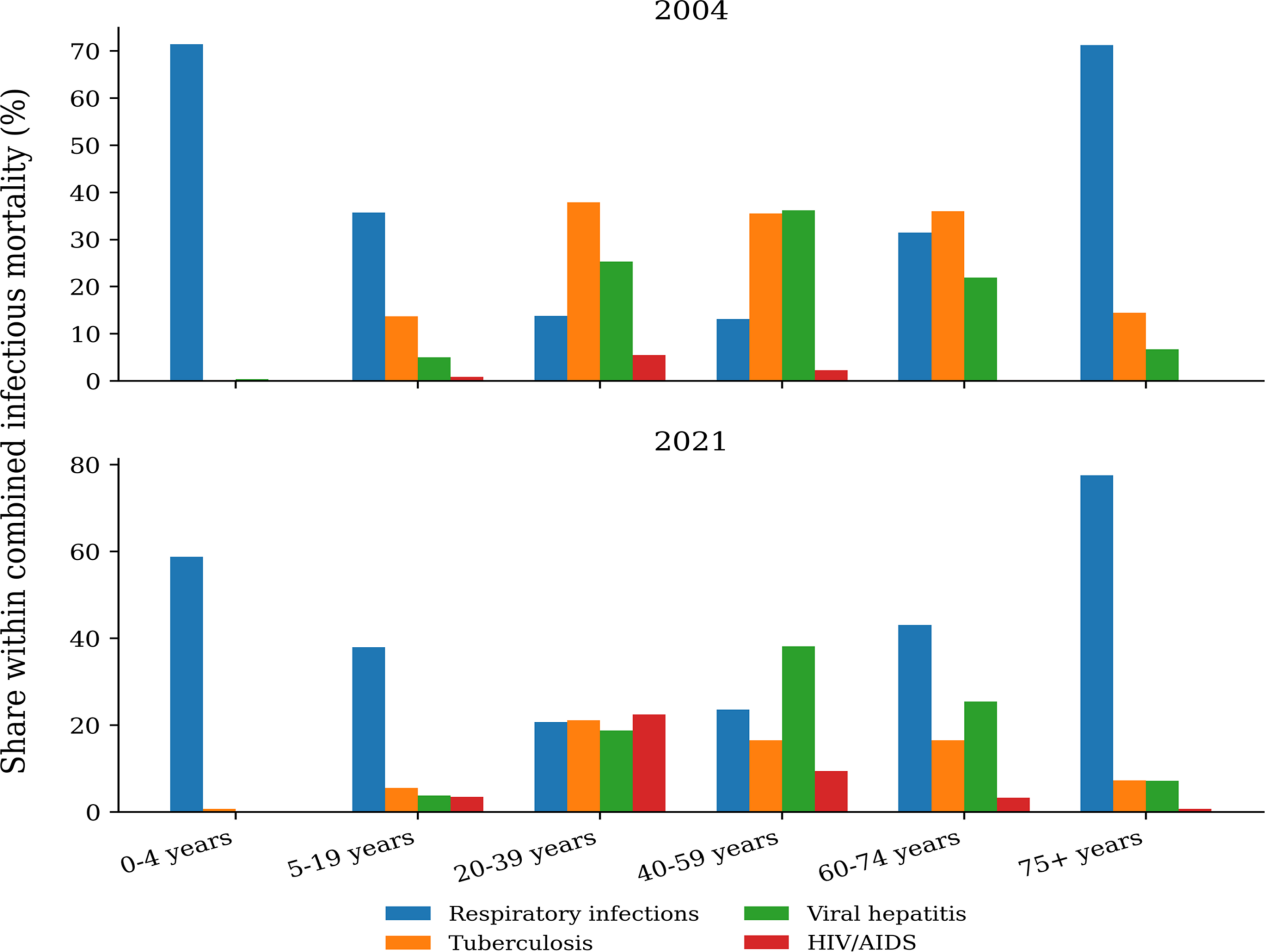
Cause composition within combined infectious mortality by age group, China, 2004 and 2021. Values are percentages within combined infectious mortality.

By 2021, for individuals aged 20 to 39, infectious diseases represented just 3.4% of all-cause mortality. The primary infectious causes of death were nearly equal in proportion: HIV/AIDS (22.49%), tuberculosis (21.16%), respiratory infections (20.71%), and viral hepatitis (18.71%), differing by less than 4 percentage points between the highest and lowest. Respiratory infections exerted a U-shaped burden, dominating both ends of the age spectrum: they accounted for 58.71% of infectious mortality among children aged 0–4 and peaked at 77.49% for those aged 75 and older. In contrast, the burden during adulthood was driven by chronic infections; HIV/AIDS emerged as the primary cause among individuals aged 20–39 (22.49%), while viral hepatitis became the predominant driver in the 40–59 age bracket, representing 38.18% of infectious deaths (Figure 3).

### HIV/AIDS: rising in older adults

The mortality burden of HIV/AIDS has shifted beyond its traditional concentration in younger populations, with the impact on older adults now equally, if not more, pronounced. Granular analysis by 5-year age intervals in 2021 revealed that the crude mortality rate among those aged 70–74 reached 0.92 per 100,000—significantly higher than the 0.21 per 100,000 observed in the 20–24 age group (Supplementary Figure S12). In broader age categories, the most striking escalation occurred among individuals aged 60–74, with an EAPC of 5.90% per year between 2013 and 2019. Notably, among all major infectious causes, HIV/AIDS exhibited the most rapid growth in mortality within the older population.

### Respiratory infections, tuberculosis, and viral hepatitis

**Figure 4:**
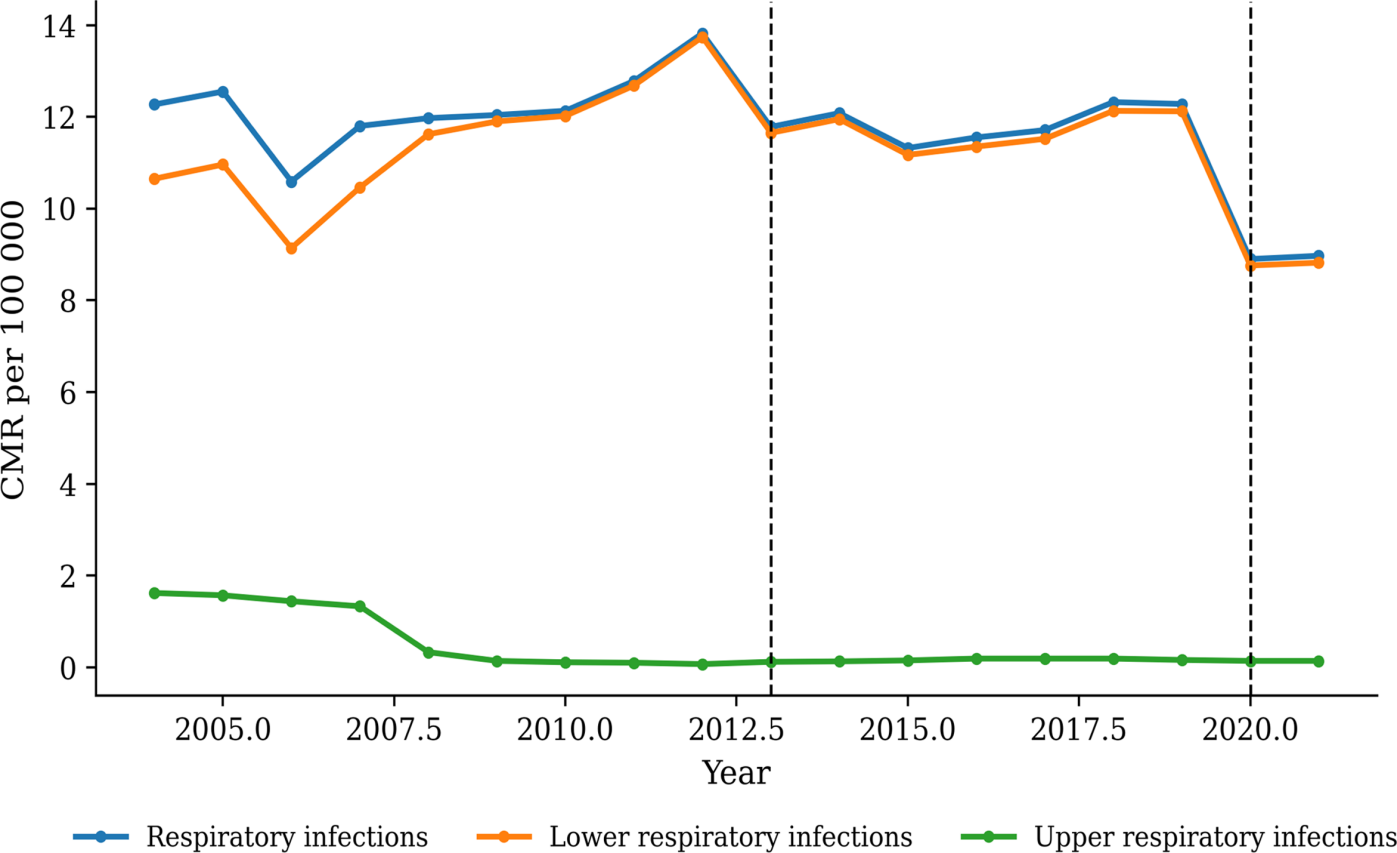
Respiratory infection mortality before and during the COVID-19 pandemic, China, 2004–2021. Shown as crude mortality rate per 100,000 population. The dashed vertical line indicates the 2013 surveillance system expansion; data before and after 2013 are not directly comparable in level. The public source does not separately code COVID-19 deaths. The 2020–2021 values are presented as observed but are not incorporated into trend estimation due to the absence of a separate COVID-19 code and potential pandemic-related changes in coding and healthcare-seeking. Sensitivity analyses for lower and upper respiratory infections are shown in Supplementary Figure S14.

Respiratory infection mortality showed a flat trend during 2013–2019 (EAPC 0.71, 95% CI –0.43 to 1.86). For 2020–2021, crude mortality rates were 8.90 and 8.97 per 100,000, respectively (Figure 4). Because the public data source does not separately code COVID-19 deaths, and because pandemic-era changes in coding, healthcare-seeking, and cause-of-death certification may have occurred, the 2020–2021 values are presented as observed and are not incorporated into trend estimation. No causal interpretation is made for this period.

Tuberculosis maintained a steady long-term decline, with an EAPC of −2.75% per year from 2013 to 2019. While viral hepatitis mortality also decreased, it remains a persistent centerpiece of the disease burden in middle age; in 2021, viral hepatitis accounted for 38.18% of all combined infectious deaths among those aged 40–59 years.

### Subgroup inequalities

In 2021, infectious disease mortality remained disproportionately high among males and in western China. HIV/AIDS exhibited the most striking subgroup disparities. The age-standardized mortality rate (ASMR) for HIV/AIDS in western China was 1.26 per 100,000 (95% CI 1.18–1.34), compared with 0.18 per 100,000 (95% CI 0.16–0.20) in eastern China, yielding a western-to-eastern ASMR ratio of 7.0 (95% CI 6.2–7.8, *p* < 0.001). Rural rates also exceeded urban rates (ASMR 0.54 vs. 0.38 per 100,000) (Supplementary Table S20).

## Discussion

**Figure 5:**
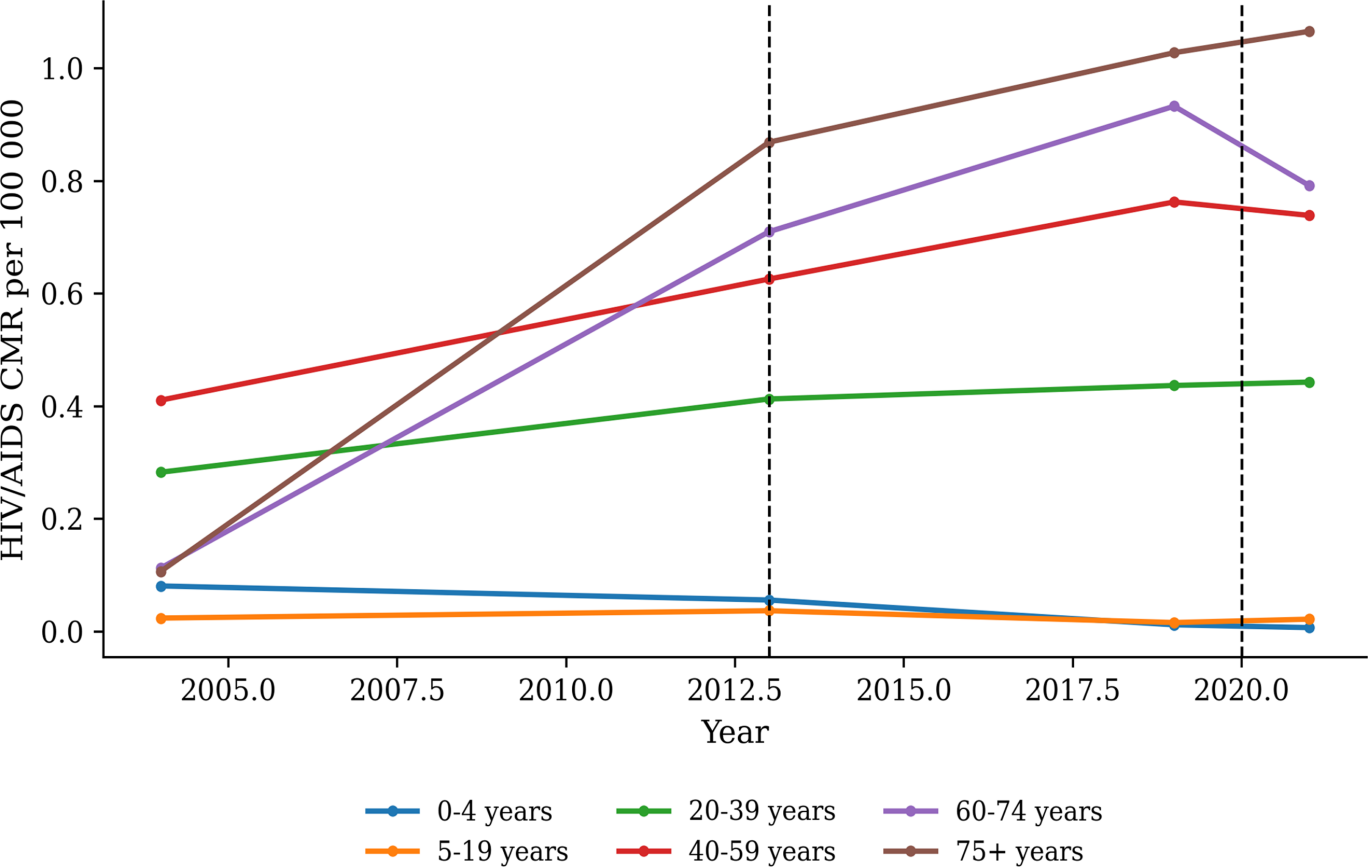
HIV/AIDS mortality by broad age group, China, 2004–2021. The clearest contemporary increases were observed at ages 60 years and older. Detailed 5-year age-band patterns are shown in Supplementary Figure S12 (e.g., CMR of 0.92 per 100,000 at ages 70–74 years in 2021).

All primary temporal inferences are based on the 2013–2019 interval; 2004–2021 trends are presented for historical context only. This national analysis demonstrates that infectious mortality in China is more accurately characterized as a profound age-specific redistribution rather than a simple residual decline. While combined infectious mortality fell substantially in absolute and age-standardized terms, this overall reduction masks a decisive demographic shift. Mortality has become increasingly concentrated among older adults, creating a distinct age profile where the dominant infectious drivers differ significantly across life stages. This transition suggests that while the historical burden of infectious disease is receding, it is being replaced by age-specific challenges that require targeted public health strategies across the lifespan. The rise in absolute infectious deaths does not indicate worsening population health; rather, it reflects population ageing and increased survival to older ages, where infectious mortality is concentrated (Figure 5).

To quantify the role of age structure in shaping the contemporary mortality burden, we compared crude mortality rates (CMR, reflecting actual age structure) with age-standardized rates (ASMR, removing age-structure effects) using 2021 data (Table 3). The age-structure contribution ranged from 24.5% in western China to 40.9% in eastern China, indicating that population age structure substantially inflates observed mortality rates across all regions. Notably, western China had the highest CMR (20.07 per 100,000) but the lowest age-structure contribution (24.5%), suggesting that its high burden is driven primarily by age-specific risk factors—such as healthcare access and comorbidity burden—rather than by age structure alone. In contrast, eastern China’s lower CMR (14.75 per 100,000) was more heavily influenced by age structure (40.9% contribution), implying that a substantial portion of its observed mortality is attributable to its older population profile. A supplementary historical Kitagawa decomposition spanning 2004–2021 (Supplementary Table S27), though limited by the 2013 surveillance expansion, showed a consistent directionality with the cross-sectional findings. These cross-sectional findings (Table 3) illustrate that the role of age structure in shaping infectious mortality burden varies markedly across regions. Importantly, a low age-structure contribution should not be misinterpreted as a low burden.

From 2013 to 2019, the overall infectious disease mortality rate remained stable (EAPC = −0.37%, with the 95% CI crossing zero), indicating a marked slowdown in the long-term declining trend. The increasing burden of respiratory infections among older adults and HIV/AIDS among middle-aged and older populations partially offset the gains achieved in tuberculosis and hepatitis control. These findings suggest a need to shift toward age-stratified strategies for infectious disease prevention and control. A supplementary cross-sectional analysis based on 2021 data (Table 3) further confirmed the above findings, showing that age structure contributed 24.5 to 40.9% of observed CMR across regions.

Several findings warrant specific consideration within the context of China’s epidemiological transition. First, respiratory infections continue to exert a high burden at both extremes of the age spectrum, remaining the primary drivers of infectious death among the youngest and oldest populations despite a generalized downward trend in national burden. Concurrently, HIV/AIDS has undergone a dual-age emergence: it is now the highest-ranked infectious cause of death among young adults and is assuming an increasingly disproportionate role in the mortality of older age cohorts. In contrast, viral hepatitis—while characterized by declining rates—persists as the structural centerpiece of infectious mortality for middle-aged adults, highlighting a distinct generational lag in disease clearance.

The tuberculosis findings align with previous research demonstrating a sustained long-run decline (11), however, they also highlight a noticeable deceleration in progress in recent years, particularly within the post-2013 surveillance data. This trend underscores the necessity for sustained investment in proactive case-finding, treatment adherence, and the strengthening of healthcare infrastructure in western China, where the disease burden remains disproportionately high, a pattern consistently observed in previous studies (10, 13, 21, 22) and also evident in our data (Table S14).

The HIV/AIDS findings deserve special emphasis. Earlier work has shown increasing HIV mortality in China, (8) suboptimal treatment outcomes in older people living with HIV (9, 14–16), and an expanding burden among older adults (23, 24). Our results extend this literature by showing that, within a national infectious-mortality framework, HIV/AIDS now exhibits a distinctive dual pattern: at ages 20–39, it is the highest-ranked infectious cause, although its proportional predominance is narrow (22.49% vs. 21.16% for tuberculosis and 20.71% for respiratory infections); and at ages 60–74, it is the fastest-growing infectious cause (EAPC 5.90% per year during 2013–2019). Our data cannot directly distinguish between the potential drivers of the increase in infections or deaths among older adults—whether due to newly acquired infections later in life or survival ageing among individuals infected at younger ages. Therefore, based solely on our data, we cannot support policy recommendations that favor one mechanism over the other. However, regardless of the underlying drivers, the observed increase in HIV/AIDS mortality among adults aged 60–74 years carries clear public health significance. Coupled with existing literature documenting late diagnosis and poor treatment outcomes among older people living with HIV in China, this trend suggests that greater attention should be paid to age-appropriate HIV testing, linkage to care, and comorbidity management for older adults. We explicitly note that these recommendations are informed by the convergence of our mortality findings with previously published evidence, rather than being derived solely from mechanistic inferences based on our data.

Our data also reveal marked disparities across subpopulations. A pronounced male predominance was observed in infectious disease mortality, particularly for HIV/AIDS (2021 male-to-female ASMR ratio: 3.90). This finding is consistent with known sex differences in healthcare-seeking behavior, occupational exposure, and the higher prevalence of high-risk behaviors among Chinese men. These results suggest that gender-specific prevention, screening, and linkage-to-care strategies may be necessary, especially in regions where the sex gap continues to widen. Regarding urban–rural differences, HIV/AIDS mortality was significantly higher in rural areas than in urban areas (ASMR: 0.54 vs. 0.38 per 100,000), consistent with delayed diagnosis and limited ART accessibility. However, overall infectious disease mortality was slightly higher in urban areas (ASMR: 10.85 vs. 9.96), primarily driven by respiratory infections, which may be associated with urbanisation-related air pollution exposure, high population density, and influenza transmission dynamics. This seemingly paradoxical pattern warrants further investigation using more detailed data on environmental exposures and healthcare resource accessibility.

Methodologically, we anchored this analysis to the 2013–2019 post-expansion baseline to ensure structural integrity. By treating 2013 as a system boundary, we isolated the contemporary trend from the systematic bias introduced during the surveillance network’s expansion. This approach effectively decoupled the actual mortality decline from the artefacts created by the 2013 expansion, which would otherwise have obscured the underlying epidemiological trajectory of the 2004–2021 series.

These results also have relevance beyond China. Similar age-related shifts in infectious mortality—deaths concentrating at the young and old ends from respiratory infections and in midlife from HIV/hepatitis—are likely to emerge in other ageing middle-income countries where tuberculosis, viral hepatitis, and HIV remain persistent burdens (2–4).

This study has several limitations that warrant consideration. First, the use of aggregated public data precludes adjustment for person-level confounders. Second, the substantial expansion of the surveillance system in 2013 introduces potential discontinuities; consequently, we have utilized the 2013–2019 interval as the primary contemporary reference. The full 2004–2021 EAPCs are provided as descriptive historical context and should not be viewed in isolation as indicators of current trajectories. Third, the lack of a distinct code for COVID-19 in the public data source complicates the interpretation of pandemic-era mortality. The decline in respiratory mortality between 2020 and 2021 could be influenced by multiple factors, including non-pharmaceutical interventions, uncertainties in cause-of-death coding, and indirect pandemic effects; our data cannot disentangle these mechanisms. Fourth, while coding transitions prior to 2009 present a minor risk of discontinuity, sensitivity analyses confirm that our primary conclusions remain robust. Finally, national-level aggregation may mask significant provincial heterogeneity.

Despite these constraints, the public health implications are clear. Infectious disease control in China has evolved beyond a single, undifferentiated agenda. Our findings identify several high-priority domains: strengthening respiratory protection for paediatric and geriatric populations, revitalizing tuberculosis control where progress has decelerated, and enhancing hepatitis detection and clinical management for middle-aged adults. The dual burden of HIV/AIDS—affecting both young adults and a rapidly growing older population—demands intensified prevention, age-specific testing, and integrated care. In particular, the persistently high HIV burden in western China and rural regions highlights critical ongoing gaps in diagnostic accessibility, effective linkage to care, and durable long-term treatment support. Addressing these divergent patterns is essential for the next phase of China’s infectious disease transition.

## Data Availability

Publicly available datasets were analyzed in this study. The data are publicly available mortality surveillance tabulations released annually by the Chinese Center for Disease Control and Prevention (China CDC) from 2004 to 2021. These data can be accessed through the China CDC official website (https://www.chinacdc.cn) and the China Public Health Science Data Center (https://www.phsciencedata.cn). No single repository or accession number is available for the entire dataset. Derived analytical tables are available from the corresponding author upon reasonable request.
