# Supplementary material for: Life-course restructuring of infectious mortality in China, 2004–2021: a national surveillance study
Source: Front Public Health. 2026 Jul 21;14:1881091. doi: 10.3389/fpubh.2026.1881091 (PMC13433531; doi:10.3389/fpubh.2026.1881091)
Supplement: Supplementary file 1 [file Supplementary_file_1.DOCX]

Supplementary appendix

# Contents

Supplementary methods

Supplementary results

Protocol synopsis

Supplementary tables (Table S1-Table S28)

Supplementary figures (Figure S1-Figure S15)

Appendix references

# Supplementary methods

**Appendix**

This appendix provides expanded methodological details, complete numerical summaries, sensitivity analyses, interaction tests, and subgroup findings that support the main manuscript. It is meant not only to document the statistical workflow but also to make the logic of the life-course restructuring argument transparent and reproducible. For contextual interpretation, we also examined Chinese-language and official Chinese sources from CNKI, WanFang Data, and the websites of the Chinese Center for Disease Control and Prevention and the National Health Commission, particularly for documentation of the surveillance system and current patterns of HIV/AIDS, tuberculosis, hepatitis, and influenza burden in China.

**Data source**

The source dataset consisted of public annual mortality surveillance tabulations for China from 2004 to 2021. The file contained deaths, monitored population denominators, and age-standardisation weights stratified by year, age, sex, residence, region, and cause. We treated the 2013 expansion of the integrated mortality surveillance system as a design boundary rather than a biological turning point, and 2020 as a pandemic-era boundary because COVID-19 was not separately coded in the public source. These decisions guided both the primary trend windows and the sensitivity analyses.

**Endpoints and definitions**

The primary endpoint was combined infectious mortality, defined as communicable and parasitic diseases plus respiratory infections. Within category 1.2, only respiratory infections were included; no other non-infectious respiratory causes were analysed. Disease-specific analyses focused on respiratory infections, tuberculosis, viral hepatitis, and HIV/AIDS. Viral hepatitis was defined as hepatitis B plus hepatitis C to preserve continuity after 2009; tuberculosis was defined using the continuous pulmonary tuberculosis code. These operational definitions were cross-checked against national Chinese technical guidance for tuberculosis control and hepatitis management.

**Outcome metrics**

Outcomes were deaths, crude mortality rates (CMRs), age-standardised mortality rates (ASMRs), and the percentage of all-cause deaths. National and subgroup ASMRs were derived by aggregating the weighted age-specific mortality components provided in the public file. We treated these metrics as complementary measures of burden. Deaths reflect service volume, CMRs reflect the burden experienced by the population, ASMRs allow better comparison across periods with different age structures, and the percentage of all-cause deaths indicates how strongly infections shape the mortality profile within each age group or population stratum.

**Statistical analysis**

Trend summaries were based on log-linear regressions of annual rates. We report estimated annual percentage changes for 2004–2021, 2004–2012, 2013–2019, and 2013–2021, along with period-on-period changes around 2019–2020 and 2020–2021 where relevant. Interaction testing assessed whether time trends differed materially across age, sex, residence, and region. For HIV/AIDS, we further examined detailed five-year age bands because broad age groups can obscure whether increases in later life are diffuse or concentrated in a single stratum.

**Purpose of the appendix**

In practical terms, the appendix addresses four questions central to interpretation. First, was the main combined endpoint defined transparently and reproducibly? Second, do the core conclusions hold across deaths, CMRs, ASMRs, and proportional mortality rather than only one metric? Third, are the main findings robust to known discontinuities such as pre-2009 coding changes and the 2020–2021 pandemic period? Fourth, does the ageing of HIV/AIDS mortality remain visible when burden is examined in more granular age bands and inequality strata?

**Supplementary results**

Across the entire period, combined infectious mortality declined in all three core metrics: CMR fell from 26.15 to 15.37 per 100,000, ASMR fell from 30.81 to 10.27, and the percentage of all-cause deaths fell from 4.32% to 2.17%. However, absolute deaths rose from 18,611 to 41,174. This contrast is not a contradiction but a key interpretive result. It shows that infectious deaths became increasingly concentrated in older populations despite declining age-adjusted risk, so the health-system burden of fatal infection did not recede as quickly as national rates alone might suggest.

The age profile of combined infectious mortality shifted strongly upward. By 2021, more than half of all combined infectious deaths occurred in people aged 75 years or older, while the contribution of ages 0–4 years had fallen sharply. Even so, the youngest age group retained a meaningful share of all-cause mortality attributable to infection, showing that redistribution did not eliminate early-life infectious relevance. The broad age tables therefore support two conclusions at once: infections increasingly became diseases of later life in absolute terms, but they also remained prominent at the start of life in relative terms.

HIV/AIDS deserves special attention because its national burden rose while its age pattern broadened. In 2004, HIV/AIDS deaths were concentrated mainly in younger and middle-aged adults; by 2021, the burden had spread further into older age groups, with particularly notable increases in the 60–74 year range. This interpretation is consistent with recent Chinese-language epidemiological analyses showing substantial growth in reported HIV infections among adults aged 60 years and older, persistent western concentration, and ongoing challenges for case finding and prevention in older adults.

Sensitivity analyses supported the robustness of the main conclusions. Lower respiratory infections closely tracked the respiratory parent category, while upper respiratory infections were rare and did not drive the observed pattern. Pre-2009 code continuity analyses showed close agreement between the older and continuous code definitions for tuberculosis and viral hepatitis. The respiratory interpretation also accords with Chinese-language analyses of influenza and respiratory disease burden in China, which emphasise the concentration of severe respiratory mortality in older adults and the need for context-specific interpretation of national respiratory trends.

A further value of the appendix is that it shows how different components of the argument connect. Tables S4–S7 establish the national multi-metric pattern; Tables S8–S15 show how redistribution and disease replacement vary by age and subgroup; Tables S18–S26 deepen the HIV/AIDS analyses; and Figures S1–S15 visualise the same patterns for rapid inspection. The appendix should therefore be read as an integrated extension of the main paper rather than a set of disconnected supporting items.

**Protocol synopsis**

**Design:** National observational study based on annual public surveillance tabulations.

**Study period:** January 1, 2004, to December 31, 2021.

**Data source:** Public mortality tabulations issued by the Chinese Center for Disease Control and Prevention.

**Primary endpoint:** Combined infectious mortality, defined as communicable and parasitic diseases plus respiratory infections; within category 1.2, only respiratory infections were included.

**Secondary endpoints:** Respiratory infections, lower respiratory infections, upper respiratory infections, tuberculosis, viral hepatitis, and HIV/AIDS.

**Primary subgroup framework:** Age (0–4, 5–19, 20–39, 40–59, 60–74, ≥75 years), sex, urban-rural residence, and region (eastern, central, western).

**Primary metrics:** Deaths, crude mortality rates, age-standardised mortality rates, and percentage of all-cause deaths.

**Primary trend windows:** 2004–2021, 2004–2012, 2013–2019, and 2013–2021; 2020–2021 interpreted separately for respiratory infections.

**Sensitivity analyses:** Exclusion of 2020–2021; respiratory parent vs lower and upper respiratory subsets; pre-2009 code continuity for tuberculosis and viral hepatitis.

**Table S1. STROBE checklist**

| **Item** | **STROBE domain** | **Recommendation summary** | **Location in manuscript** |
| --- | --- | --- | --- |
| 1 | Title and abstract | Indicate study design and balanced summary | Main manuscript title page and Summary |
| 2 | Background/rationale | Explain scientific background and rationale | Introduction paragraphs 1-3 |
| 3 | Objectives | State specific objectives | Introduction paragraph 3 |
| 4 | Study design | Present key elements of study design | Methods - Data source and study design |
| 5 | Setting | Describe setting, locations, dates | Methods - Data source and study design |
| 6 | Participants | Describe eligibility/source of participants | Not applicable; public aggregated surveillance tabulations |
| 7 | Variables | Define outcomes, exposures, confounders | Methods - Outcomes, age grouping, and statistical analysis |
| 8 | Data sources/measurement | Describe sources and methods of assessment | Methods and appendix coding framework |
| 9 | Bias | Describe efforts to address potential bias | Methods and Discussion limitations |
| 10 | Study size | Explain how study size was arrived at | Entire public annual surveillance dataset, 2004-2021 |
| 11 | Quantitative variables | Explain handling of quantitative variables | Methods and appendix statistical analysis |
| 12 | Statistical methods | Describe statistical methods | Methods and supplementary methods |
| 13 | Participants/results | Report numbers at each stage | Table 1, appendix tables, figures |
| 14 | Descriptive data | Give characteristics of study participants | Appendix tables by age, sex, residence, region |
| 15 | Outcome data | Report numbers or summary measures | Main results, tables, figures |
| 16 | Main results | Give estimates and precision | Main results and appendix trend tables |
| 17 | Other analyses | Report subgroup/sensitivity analyses | Main text and appendix sensitivity analyses |
| 18 | Key results | Summarise key results with reference to objectives | Discussion paragraph 1 |
| 19 | Limitations | Discuss limitations | Discussion paragraph 5 |
| 20 | Interpretation | Give cautious interpretation | Discussion paragraphs 2-6 |
| 21 | Generalisability | Discuss generalisability | Discussion paragraphs 5-6 |
| 22 | Funding | Give funding source and role | Title page and Methods - Role of funding source |

**Table S2. Search strategy and selection criteria for the research-in-context review**

| **Field** | **Specification** |
| --- | --- |
| Databases and sources | PubMed, Web of Science, Google Scholar, and reference lists of retrieved papers |
| Search end date | April 8, 2026 |
| Language strategy | English-language and Chinese-language publications were considered; key Chinese surveillance reports were retained when directly relevant |
| Core concepts | China; mortality; infectious disease; respiratory infection; tuberculosis; viral hepatitis; hepatitis B; hepatitis C; HIV; AIDS; surveillance; age-period-cohort; life course |
| Eligibility | National or subnational mortality studies, surveillance analyses, age-period-cohort analyses, and disease-specific mortality reports relevant to respiratory infections, tuberculosis, viral hepatitis, or HIV/AIDS |
| Exclusions | Reports unrelated to mortality, duplicate publications, purely clinical case series without mortality data, and papers not informing the Chinese life-course infectious mortality context |

**Table S3. Analytic definitions and surveillance coding framework**

| **Analytic category** | **Operational definition** | **Use in analysis** |
| --- | --- | --- |
| Combined infectious mortality | 1.1.0.0.A communicable and parasitic diseases + 1.2.0.0.B respiratory infections | Primary endpoint |
| Respiratory infections | 1.2.0.0.B | Primary disease category; interpreted separately in 2020-2021 |
| Lower respiratory infections | 1.2.1.0.1 | Respiratory sensitivity analysis |
| Upper respiratory infections | 1.2.2.0.2 | Respiratory sensitivity analysis |
| Tuberculosis | 1.1.1.0.1 pulmonary tuberculosis | Primary tuberculosis definition |
| Respiratory tuberculosis (pre-2009 code) | 1.1.1.1.(09 post)a | Sensitivity analysis only |
| Viral hepatitis | 1.1.7.0.7 hepatitis B + 1.1.8.0.8 hepatitis C | Primary viral hepatitis definition |
| Viral hepatitis (pre-2009 code) | 1.1.7.0.(09prev) | Sensitivity analysis only |
| HIV/AIDS | 1.1.3.0.3 | Primary HIV/AIDS definition |
| Negligible parasitic/tropical causes | Malaria, tropical diseases, schistosomiasis, dengue, trichiniasis | Excluded from disease-decomposition emphasis because mortality was persistently negligible |

**Table S4. National annual burden of combined infectious mortality and the four major component causes, 2004-2021**

| **Year** | **Disease** | **Deaths** | **CMR (/100 000)** | **ASMR (/100 000)** | **Share of all-cause deaths (%)** |
| --- | --- | --- | --- | --- | --- |
| 2004 | Combined infectious mortality | 18611 | 26.15 | 30.81 | 4.32 |
| 2005 | Combined infectious mortality | 18921 | 26.47 | 31.04 | 4.32 |
| 2006 | Combined infectious mortality | 14169 | 21.46 | 24.86 | 4.08 |
| 2007 | Combined infectious mortality | 15268 | 21.36 | 24.22 | 3.81 |
| 2008 | Combined infectious mortality | 15565 | 21.05 | 23.30 | 3.67 |
| 2009 | Combined infectious mortality | 15298 | 20.39 | 24.56 | 3.50 |
| 2010 | Combined infectious mortality | 15803 | 20.06 | 25.06 | 3.49 |
| 2011 | Combined infectious mortality | 15553 | 20.10 | 23.47 | 3.49 |
| 2012 | Combined infectious mortality | 16105 | 20.86 | 19.85 | 3.50 |
| 2013 | Combined infectious mortality | 44785 | 19.71 | 17.60 | 3.06 |
| 2014 | Combined infectious mortality | 50672 | 19.98 | 17.34 | 3.08 |
| 2015 | Combined infectious mortality | 49284 | 19.13 | 16.56 | 2.95 |
| 2016 | Combined infectious mortality | 51094 | 19.30 | 15.66 | 2.93 |
| 2017 | Combined infectious mortality | 51927 | 19.15 | 15.35 | 2.91 |
| 2018 | Combined infectious mortality | 53179 | 19.53 | 14.96 | 2.92 |
| 2019 | Combined infectious mortality | 53491 | 19.32 | 15.04 | 2.86 |
| 2020 | Combined infectious mortality | 43002 | 15.44 | 11.25 | 2.28 |
| 2021 | Combined infectious mortality | 41174 | 15.37 | 10.27 | 2.17 |
| 2004 | Respiratory infections | 8733 | 12.27 | 14.86 | 2.03 |
| 2005 | Respiratory infections | 8969 | 12.55 | 15.04 | 2.05 |
| 2006 | Respiratory infections | 6988 | 10.59 | 12.57 | 2.01 |
| 2007 | Respiratory infections | 8435 | 11.80 | 13.62 | 2.10 |
| 2008 | Respiratory infections | 8846 | 11.97 | 13.55 | 2.08 |
| 2009 | Respiratory infections | 9033 | 12.04 | 15.71 | 2.06 |
| 2010 | Respiratory infections | 9555 | 12.13 | 16.71 | 2.11 |
| 2011 | Respiratory infections | 9890 | 12.78 | 16.09 | 2.22 |
| 2012 | Respiratory infections | 10670 | 13.82 | 13.13 | 2.32 |
| 2013 | Respiratory infections | 26776 | 11.78 | 10.38 | 1.83 |
| 2014 | Respiratory infections | 30645 | 12.08 | 10.22 | 1.86 |
| 2015 | Respiratory infections | 29166 | 11.32 | 9.55 | 1.74 |
| 2016 | Respiratory infections | 30570 | 11.55 | 8.97 | 1.75 |
| 2017 | Respiratory infections | 31749 | 11.71 | 8.97 | 1.78 |
| 2018 | Respiratory infections | 33553 | 12.32 | 8.94 | 1.84 |
| 2019 | Respiratory infections | 34005 | 12.28 | 9.29 | 1.82 |
| 2020 | Respiratory infections | 24795 | 8.90 | 6.15 | 1.31 |
| 2021 | Respiratory infections | 24026 | 8.97 | 5.46 | 1.26 |
| 2004 | Tuberculosis | 4314 | 6.06 | 7.05 | 1.00 |
| 2005 | Tuberculosis | 4415 | 6.18 | 7.18 | 1.01 |
| 2006 | Tuberculosis | 2758 | 4.18 | 4.73 | 0.79 |
| 2007 | Tuberculosis | 2707 | 3.79 | 4.22 | 0.68 |
| 2008 | Tuberculosis | 2523 | 3.41 | 3.68 | 0.59 |
| 2009 | Tuberculosis | 2486 | 3.31 | 3.53 | 0.57 |
| 2010 | Tuberculosis | 2486 | 3.16 | 3.37 | 0.55 |
| 2011 | Tuberculosis | 2332 | 3.01 | 3.01 | 0.52 |
| 2012 | Tuberculosis | 2312 | 2.99 | 2.84 | 0.50 |
| 2013 | Tuberculosis | 5765 | 2.54 | 2.29 | 0.39 |
| 2014 | Tuberculosis | 5885 | 2.32 | 2.07 | 0.36 |
| 2015 | Tuberculosis | 6024 | 2.34 | 2.08 | 0.36 |
| 2016 | Tuberculosis | 6134 | 2.32 | 1.96 | 0.35 |
| 2017 | Tuberculosis | 5905 | 2.18 | 1.83 | 0.33 |
| 2018 | Tuberculosis | 5958 | 2.19 | 1.78 | 0.33 |
| 2019 | Tuberculosis | 5767 | 2.08 | 1.68 | 0.31 |
| 2020 | Tuberculosis | 5147 | 1.85 | 1.42 | 0.27 |
| 2021 | Tuberculosis | 4628 | 1.73 | 1.25 | 0.24 |
| 2004 | Viral hepatitis (HBV+HCV) | 2955 | 4.15 | 4.81 | 0.69 |
| 2005 | Viral hepatitis (HBV+HCV) | 2732 | 3.82 | 4.45 | 0.62 |
| 2006 | Viral hepatitis (HBV+HCV) | 2510 | 3.80 | 4.33 | 0.72 |
| 2007 | Viral hepatitis (HBV+HCV) | 2128 | 2.98 | 3.33 | 0.53 |
| 2008 | Viral hepatitis (HBV+HCV) | 2318 | 3.14 | 3.38 | 0.55 |
| 2009 | Viral hepatitis (HBV+HCV) | 2024 | 2.70 | 2.84 | 0.46 |
| 2010 | Viral hepatitis (HBV+HCV) | 1773 | 2.25 | 2.34 | 0.39 |
| 2011 | Viral hepatitis (HBV+HCV) | 1666 | 2.15 | 2.12 | 0.37 |
| 2012 | Viral hepatitis (HBV+HCV) | 1580 | 2.05 | 1.94 | 0.34 |
| 2013 | Viral hepatitis (HBV+HCV) | 6435 | 2.83 | 2.55 | 0.44 |
| 2014 | Viral hepatitis (HBV+HCV) | 7649 | 3.02 | 2.68 | 0.47 |
| 2015 | Viral hepatitis (HBV+HCV) | 7963 | 3.09 | 2.75 | 0.48 |
| 2016 | Viral hepatitis (HBV+HCV) | 8287 | 3.13 | 2.68 | 0.48 |
| 2017 | Viral hepatitis (HBV+HCV) | 8144 | 3.00 | 2.56 | 0.46 |
| 2018 | Viral hepatitis (HBV+HCV) | 7427 | 2.73 | 2.28 | 0.41 |
| 2019 | Viral hepatitis (HBV+HCV) | 7535 | 2.72 | 2.18 | 0.40 |
| 2020 | Viral hepatitis (HBV+HCV) | 7454 | 2.68 | 2.06 | 0.40 |
| 2021 | Viral hepatitis (HBV+HCV) | 6873 | 2.57 | 1.91 | 0.36 |
| 2004 | HIV/AIDS | 158 | 0.22 | 0.23 | 0.04 |
| 2005 | HIV/AIDS | 186 | 0.26 | 0.28 | 0.04 |
| 2006 | HIV/AIDS | 155 | 0.23 | 0.25 | 0.04 |
| 2007 | HIV/AIDS | 232 | 0.32 | 0.33 | 0.06 |
| 2008 | HIV/AIDS | 206 | 0.28 | 0.29 | 0.05 |
| 2009 | HIV/AIDS | 218 | 0.29 | 0.30 | 0.05 |
| 2010 | HIV/AIDS | 338 | 0.43 | 0.43 | 0.07 |
| 2011 | HIV/AIDS | 262 | 0.34 | 0.33 | 0.06 |
| 2012 | HIV/AIDS | 281 | 0.36 | 0.35 | 0.06 |
| 2013 | HIV/AIDS | 1029 | 0.45 | 0.44 | 0.07 |
| 2014 | HIV/AIDS | 1204 | 0.47 | 0.46 | 0.07 |
| 2015 | HIV/AIDS | 1236 | 0.48 | 0.47 | 0.07 |
| 2016 | HIV/AIDS | 1404 | 0.53 | 0.51 | 0.08 |
| 2017 | HIV/AIDS | 1488 | 0.55 | 0.53 | 0.08 |
| 2018 | HIV/AIDS | 1510 | 0.55 | 0.53 | 0.08 |
| 2019 | HIV/AIDS | 1511 | 0.55 | 0.50 | 0.08 |
| 2020 | HIV/AIDS | 1380 | 0.50 | 0.45 | 0.07 |
| 2021 | HIV/AIDS | 1406 | 0.52 | 0.48 | 0.07 |

**Table S5. CMR trend summaries by disease**

| **Disease** | **2004-2021** | **2004-2012** | **2013-2019** | **2013-2021** | **2019→2020 change (%)** |
| --- | --- | --- | --- | --- | --- |
| Combined infectious mortality | -2.15 (-2.76 to -1.53) | -3.14 (-4.77 to -1.48) | -0.37 (-0.93 to 0.19) | -2.85 (-4.59 to -1.08) | -20.08 |
| Respiratory infections | -0.95 (-1.84 to -0.05) | 1.38 (-0.26 to 3.05) | 0.71 (-0.43 to 1.86) | -2.92 (-5.48 to -0.30) | -27.51 |
| Tuberculosis | -6.34 (-7.27 to -5.41) | -9.02 (-11.73 to -6.22) | -2.75 (-3.60 to -1.89) | -4.10 (-5.14 to -3.04) | -11.28 |
| Viral hepatitis (HBV+HCV) | -1.56 (-3.08 to -0.02) | -9.06 (-10.38 to -7.72) | -1.24 (-3.29 to 0.86) | -1.89 (-3.16 to -0.61) | -1.66 |
| HIV/AIDS | 5.65 (4.50 to 6.81) | 6.65 (3.25 to 10.16) | 3.65 (2.40 to 4.92) | 1.72 (0.17 to 3.29) | -9.21 |

*Note:EAPC = estimated annual percentage change based on log-linear regression of annual CMRs.CRITICAL NOTE: The mortality surveillance system expanded from 161 to 605 points in 2013. Therefore, the 2004–2021 and 2004–2012 EAPCs are provided for descriptive historical context only and should not be directly compared with post-2013 EAPCs. Primary trend inferences in the main manuscript are based exclusively on the 2013–2019 period. The 2013–2021 column is also affected by pandemic-era uncertainties and is provided for completeness.*

**Table S6. ASMR trend summaries by disease**

| **Disease** | **2004-2021** | **2004-2012** | **2013-2019** | **2013-2021** | **2019→2020 change (%)** |
| --- | --- | --- | --- | --- | --- |
| Combined infectious mortality | -5.59 (-6.29 to -4.88) | -4.19 (-6.10 to -2.24) | -2.96 (-3.65 to -2.27) | -5.96 (-8.03 to -3.86) | -25.18 |
| Respiratory infections | -5.13 (-6.52 to -3.72) | 0.70 (-1.90 to 3.38) | -2.34 (-3.80 to -0.86) | -6.69 (-9.70 to -3.58) | -33.88 |
| Tuberculosis | -8.96 (-9.83 to -8.08) | -11.15 (-13.65 to -8.58) | -4.76 (-5.52 to -3.99) | -6.59 (-7.94 to -5.23) | -15.59 |
| Viral hepatitis (HBV+HCV) | -4.19 (-5.65 to -2.70) | -11.38 (-12.65 to -10.09) | -3.00 (-5.25 to -0.69) | -4.15 (-5.67 to -2.61) | -5.56 |
| HIV/AIDS | 4.72 (3.56 to 5.89) | 5.53 (2.17 to 8.99) | 2.91 (1.26 to 4.60) | 0.81 (-1.01 to 2.66) | -10.94 |

*Note: EAPC = estimated annual percentage change based on log-linear regression of annual ASMRs. CRITICAL NOTE: The mortality surveillance system expanded from 161 to 605 points in 2013. Therefore, the 2004–2021 and 2004–2012 EAPCs are provided for descriptive historical context only and should not be directly compared with post-2013 EAPCs. Primary trend inferences in the main manuscript are based exclusively on the 2013–2019 period. The 2013–2021 column is also affected by pandemic-era uncertainties and is provided for completeness. 2019→2020 change (%) = percentage change in ASMR from 2019 to 2020; these values are presented as observed but are not incorporated into trend estimation due to the absence of a separate COVID-19 code and potential pandemic-related changes.*

**Table S7. Trend summaries for the percentage of all-cause deaths**

| **Disease** | **2004-2021** | **2004-2012** | **2013-2019** | **2013-2021** | **2019→2020 change (%)** |
| --- | --- | --- | --- | --- | --- |
| Combined infectious mortality | -3.40 (-3.89 to -2.91) | -3.09 (-3.94 to -2.22) | -1.14 (-1.59 to -0.67) | -3.84 (-5.69 to -1.95) | -20.41 |
| Respiratory infections | -2.22 (-3.17 to -1.26) | 1.43 (0.77 to 2.10) | -0.06 (-1.10 to 0.98) | -3.91 (-6.56 to -1.19) | -27.81 |
| Tuberculosis | -7.55 (-8.28 to -6.80) | -8.97 (-10.96 to -6.93) | -3.49 (-4.29 to -2.69) | -5.07 (-6.27 to -3.85) | -11.64 |
| Viral hepatitis (HBV+HCV) | -2.83 (-4.15 to -1.48) | -9.01 (-10.98 to -7.01) | -2.00 (-4.13 to 0.18) | -2.89 (-4.30 to -1.46) | -2.06 |
| HIV/AIDS | 4.29 (3.12 to 5.48) | 6.70 (3.20 to 10.33) | 2.86 (1.56 to 4.18) | 0.68 (-0.97 to 2.37) | -9.58 |

*Note: EAPC = estimated annual percentage change based on log-linear regression of annual percentages of all-cause deaths. CRITICAL NOTE: The mortality surveillance system expanded from 161 to 605 points in 2013. Therefore, the 2004–2021 and 2004–2012 EAPCs are provided for descriptive historical context only and should not be directly compared with post-2013 EAPCs. Primary trend inferences in the main manuscript are based exclusively on the 2013–2019 period. The 2013–2021 column is also affected by pandemic-era uncertainties and is provided for completeness. 2019→2020 change (%) = percentage change from 2019 to 2020; these values are presented as observed but are not incorporated into trend estimation due to the absence of a separate COVID-19 code and potential pandemic-related changes.*

**Table S8. Combined infectious mortality by broad age group in 2004 and 2021**

| **Year** | **Age group** | **Deaths** | **CMR (/100 000)** | **Share of all-cause deaths (%)** |
| --- | --- | --- | --- | --- |
| 2004 | 0-4 years | 2186 | 58.78 | 21.08 |
| 2004 | 20-39 years | 1289 | 5.13 | 4.87 |
| 2004 | 40-59 years | 3137 | 18.14 | 4.00 |
| 2004 | 5-19 years | 459 | 2.71 | 7.03 |
| 2004 | 60-74 years | 4530 | 72.91 | 3.19 |
| 2004 | 75+ years | 7010 | 373.50 | 4.19 |
| 2021 | 0-4 years | 557 | 3.82 | 7.97 |
| 2021 | 20-39 years | 1347 | 1.97 | 3.44 |
| 2021 | 40-59 years | 6577 | 7.87 | 2.56 |
| 2021 | 5-19 years | 287 | 0.62 | 3.08 |
| 2021 | 60-74 years | 9743 | 23.88 | 1.74 |
| 2021 | 75+ years | 22663 | 160.02 | 2.20 |

**Table S9. Age redistribution of combined infectious deaths in 2004 and 2021**

| **Year** | **Age group** | **Deaths** | **Share of combined infectious deaths (%)** |
| --- | --- | --- | --- |
| 2004 | 0-4 years | 2186 | 11.75 |
| 2004 | 20-39 years | 1289 | 6.93 |
| 2004 | 40-59 years | 3137 | 16.86 |
| 2004 | 5-19 years | 459 | 2.47 |
| 2004 | 60-74 years | 4530 | 24.34 |
| 2004 | 75+ years | 7010 | 37.67 |
| 2021 | 0-4 years | 557 | 1.35 |
| 2021 | 20-39 years | 1347 | 3.27 |
| 2021 | 40-59 years | 6577 | 15.97 |
| 2021 | 5-19 years | 287 | 0.70 |
| 2021 | 60-74 years | 9743 | 23.66 |
| 2021 | 75+ years | 22663 | 55.04 |

**Table S10. Cause composition within combined infectious mortality by broad age group in 2004**

| **Age group** | **Disease** | **Share within combined infectious mortality (%)** |
| --- | --- | --- |
| 0-4 years | Respiratory infections | 71.41 |
| 0-4 years | Tuberculosis | 0.18 |
| 0-4 years | Viral hepatitis (HBV+HCV) | 0.37 |
| 0-4 years | HIV/AIDS | 0.14 |
| 5-19 years | Respiratory infections | 35.73 |
| 5-19 years | Tuberculosis | 13.73 |
| 5-19 years | Viral hepatitis (HBV+HCV) | 5.01 |
| 5-19 years | HIV/AIDS | 0.87 |
| 20-39 years | Respiratory infections | 13.81 |
| 20-39 years | Tuberculosis | 37.94 |
| 20-39 years | Viral hepatitis (HBV+HCV) | 25.37 |
| 20-39 years | HIV/AIDS | 5.51 |
| 40-59 years | Respiratory infections | 13.17 |
| 40-59 years | Tuberculosis | 35.51 |
| 40-59 years | Viral hepatitis (HBV+HCV) | 36.21 |
| 40-59 years | HIV/AIDS | 2.26 |
| 60-74 years | Respiratory infections | 31.43 |
| 60-74 years | Tuberculosis | 35.98 |
| 60-74 years | Viral hepatitis (HBV+HCV) | 21.90 |
| 60-74 years | HIV/AIDS | 0.15 |
| 75+ years | Respiratory infections | 71.23 |
| 75+ years | Tuberculosis | 14.47 |
| 75+ years | Viral hepatitis (HBV+HCV) | 6.69 |
| 75+ years | HIV/AIDS | 0.03 |

**Table S11. Cause composition within combined infectious mortality by broad age group in 2021**

| **Age group** | **Disease** | **Share within combined infectious mortality (%)** |
| --- | --- | --- |
| 0-4 years | Respiratory infections | 58.71 |
| 0-4 years | Tuberculosis | 0.72 |
| 0-4 years | Viral hepatitis (HBV+HCV) | 0.00 |
| 0-4 years | HIV/AIDS | 0.18 |
| 5-19 years | Respiratory infections | 37.98 |
| 5-19 years | Tuberculosis | 5.57 |
| 5-19 years | Viral hepatitis (HBV+HCV) | 3.83 |
| 5-19 years | HIV/AIDS | 3.48 |
| 20-39 years | Respiratory infections | 20.71 |
| 20-39 years | Tuberculosis | 21.16 |
| 20-39 years | Viral hepatitis (HBV+HCV) | 18.71 |
| 20-39 years | HIV/AIDS | 22.49 |
| 40-59 years | Respiratory infections | 23.58 |
| 40-59 years | Tuberculosis | 16.47 |
| 40-59 years | Viral hepatitis (HBV+HCV) | 38.18 |
| 40-59 years | HIV/AIDS | 9.40 |
| 60-74 years | Respiratory infections | 43.09 |
| 60-74 years | Tuberculosis | 16.46 |
| 60-74 years | Viral hepatitis (HBV+HCV) | 25.39 |
| 60-74 years | HIV/AIDS | 3.32 |
| 75+ years | Respiratory infections | 77.49 |
| 75+ years | Tuberculosis | 7.22 |
| 75+ years | Viral hepatitis (HBV+HCV) | 7.17 |
| 75+ years | HIV/AIDS | 0.67 |

**Table S12. Sex-specific burden in 2021**

| **Disease** | **Group** | **Deaths** | **CMR** | **ASMR** | **Share of all-cause deaths (%)** |
| --- | --- | --- | --- | --- | --- |
| Combined infectious mortality | Female | 15451 | 11.72 | 6.72 | 1.92 |
| Combined infectious mortality | Male | 25723 | 18.91 | 14.18 | 2.35 |
| Respiratory infections | Female | 10189 | 7.73 | 4.02 | 1.27 |
| Respiratory infections | Male | 13837 | 10.17 | 7.16 | 1.26 |
| Tuberculosis | Female | 1065 | 0.81 | 0.54 | 0.13 |
| Tuberculosis | Male | 3563 | 2.62 | 2.02 | 0.32 |
| Viral hepatitis (HBV+HCV) | Female | 2165 | 1.64 | 1.06 | 0.27 |
| Viral hepatitis (HBV+HCV) | Male | 4708 | 3.46 | 2.78 | 0.43 |
| HIV/AIDS | Female | 284 | 0.22 | 0.20 | 0.04 |
| HIV/AIDS | Male | 1122 | 0.82 | 0.78 | 0.10 |

**Table S13. Urban-rural burden in 2021**

| **Disease** | **Group** | **Deaths** | **CMR** | **ASMR** | **Share of all-cause deaths (%)** |
| --- | --- | --- | --- | --- | --- |
| Combined infectious mortality | Rural | 25412 | 14.43 | 9.96 | 1.94 |
| Combined infectious mortality | Urban | 15762 | 17.18 | 10.85 | 2.66 |
| Respiratory infections | Rural | 13390 | 7.60 | 4.74 | 1.02 |
| Respiratory infections | Urban | 10636 | 11.59 | 6.79 | 1.80 |
| Tuberculosis | Rural | 3316 | 1.88 | 1.38 | 0.25 |
| Tuberculosis | Urban | 1312 | 1.43 | 1.00 | 0.22 |
| Viral hepatitis (HBV+HCV) | Rural | 4910 | 2.79 | 2.10 | 0.37 |
| Viral hepatitis (HBV+HCV) | Urban | 1963 | 2.14 | 1.57 | 0.33 |
| HIV/AIDS | Rural | 994 | 0.56 | 0.54 | 0.08 |
| HIV/AIDS | Urban | 412 | 0.45 | 0.38 | 0.07 |

**Table S14. Region-specific burden in 2021**

| **Disease** | **Group** | **Deaths** | **CMR** | **ASMR** | **Share of all-cause deaths (%)** |
| --- | --- | --- | --- | --- | --- |
| Combined infectious mortality | Eastern | 15811 | 14.75 | 8.72 | 2.10 |
| Combined infectious mortality | Central | 11112 | 12.39 | 8.47 | 1.74 |
| Combined infectious mortality | Western | 14251 | 20.07 | 15.16 | 2.80 |
| Respiratory infections | Eastern | 10867 | 10.14 | 5.51 | 1.44 |
| Respiratory infections | Central | 5875 | 6.55 | 4.09 | 0.92 |
| Respiratory infections | Western | 7284 | 10.26 | 7.13 | 1.43 |
| Tuberculosis | Eastern | 1306 | 1.22 | 0.82 | 0.17 |
| Tuberculosis | Central | 1369 | 1.53 | 1.11 | 0.21 |
| Tuberculosis | Western | 1953 | 2.75 | 2.15 | 0.38 |
| Viral hepatitis (HBV+HCV) | Eastern | 1837 | 1.71 | 1.22 | 0.24 |
| Viral hepatitis (HBV+HCV) | Central | 2399 | 2.68 | 1.99 | 0.38 |
| Viral hepatitis (HBV+HCV) | Western | 2637 | 3.71 | 2.99 | 0.52 |
| HIV/AIDS | Eastern | 201 | 0.19 | 0.18 | 0.03 |
| HIV/AIDS | Central | 245 | 0.27 | 0.26 | 0.04 |
| HIV/AIDS | Western | 960 | 1.35 | 1.26 | 0.19 |

*Note: CMR = crude mortality rate (per 100,000 population). ASMR = age-standardised mortality rate (per 100,000 population). Share = percentage of all-cause deaths. For HIV/AIDS, the western-to-eastern ASMR ratio was 7.0 (95% CI 6.2–7.8, p < 0.001). ASMR differences were greatest for western versus eastern China and for male versus female populations; rural rates also exceeded urban rates (see Table S13).*

**Table S15. Broad-age EAPCs for the five primary endpoints**

| **Disease** | **Age group** | **EAPC 2004-2021** | **EAPC 2013-2019** |
| --- | --- | --- | --- |
| Combined infectious mortality | 0-4 years | -14.50 (-15.99 to -12.97) | -14.70 (-16.96 to -12.38) |
| Combined infectious mortality | 5-19 years | -6.82 (-7.86 to -5.76) | -5.04 (-7.85 to -2.15) |
| Combined infectious mortality | 20-39 years | -4.96 (-5.54 to -4.37) | -3.11 (-4.48 to -1.71) |
| Combined infectious mortality | 40-59 years | -4.30 (-5.40 to -3.19) | -1.39 (-2.24 to -0.53) |
| Combined infectious mortality | 60-74 years | -5.14 (-5.99 to -4.29) | -3.07 (-4.62 to -1.48) |
| Combined infectious mortality | 75+ years | -4.12 (-4.95 to -3.28) | -1.62 (-3.66 to 0.46) |
| Respiratory infections | 0-4 years | -15.48 (-17.24 to -13.67) | -14.69 (-16.58 to -12.76) |
| Respiratory infections | 5-19 years | -5.85 (-7.37 to -4.30) | -2.95 (-6.54 to 0.78) |
| Respiratory infections | 20-39 years | -1.73 (-2.82 to -0.63) | 0.91 (-3.52 to 5.55) |
| Respiratory infections | 40-59 years | -0.38 (-1.51 to 0.76) | 1.63 (-2.65 to 6.10) |
| Respiratory infections | 60-74 years | -3.45 (-4.14 to -2.75) | -1.88 (-3.66 to -0.07) |
| Respiratory infections | 75+ years | -3.75 (-4.84 to -2.64) | -1.39 (-3.42 to 0.69) |
| Tuberculosis | 0-4 years | -13.03 (-17.30 to -8.54) | -16.87 (-21.18 to -12.33) |
| Tuberculosis | 5-19 years | -11.29 (-13.05 to -9.50) | -10.00 (-16.06 to -3.49) |
| Tuberculosis | 20-39 years | -8.25 (-9.09 to -7.40) | -3.88 (-6.04 to -1.67) |
| Tuberculosis | 40-59 years | -8.63 (-9.57 to -7.69) | -4.48 (-5.39 to -3.57) |
| Tuberculosis | 60-74 years | -9.71 (-10.72 to -8.69) | -5.90 (-8.04 to -3.70) |
| Tuberculosis | 75+ years | -8.00 (-9.14 to -6.85) | -2.83 (-4.96 to -0.66) |
| Viral hepatitis (HBV+HCV) | 0-4 years | -12.89 (-16.32 to -9.33) | -16.76 (-33.24 to 3.79) |
| Viral hepatitis (HBV+HCV) | 5-19 years | -10.30 (-12.61 to -7.92) | -11.95 (-20.76 to -2.17) |
| Viral hepatitis (HBV+HCV) | 20-39 years | -6.79 (-8.02 to -5.54) | -6.79 (-8.42 to -5.13) |
| Viral hepatitis (HBV+HCV) | 40-59 years | -4.16 (-5.67 to -2.63) | -1.83 (-3.39 to -0.24) |
| Viral hepatitis (HBV+HCV) | 60-74 years | -3.84 (-5.51 to -2.14) | -3.16 (-6.65 to 0.45) |
| Viral hepatitis (HBV+HCV) | 75+ years | -2.79 (-4.43 to -1.12) | -2.19 (-5.66 to 1.42) |
| HIV/AIDS | 0-4 years | -9.73 (-14.54 to -4.64) | -14.60 (-28.92 to 2.59) |
| HIV/AIDS | 5-19 years | -1.63 (-5.20 to 2.07) | -6.77 (-20.09 to 8.78) |
| HIV/AIDS | 20-39 years | 2.73 (1.04 to 4.46) | 1.33 (-2.05 to 4.82) |
| HIV/AIDS | 40-59 years | 3.67 (2.61 to 4.74) | 3.47 (1.73 to 5.24) |
| HIV/AIDS | 60-74 years | 11.92 (9.56 to 14.33) | 5.90 (4.36 to 7.46) |
| HIV/AIDS | 75+ years | 17.20 (12.61 to 21.99) | 6.03 (-2.33 to 15.10) |

*Note: EAPC = estimated annual percentage change. Due to the 2013 surveillance system expansion, the 2004–2021 EAPCs are provided for descriptive historical context only and are not directly comparable with the 2013–2019 EAPCs. Primary inferences about contemporary age-specific trends should be based on the 2013–2019 column. Estimates with wide confidence intervals (e.g., HIV/AIDS at ages 75+ years) should be interpreted with caution.*

**Table S16. Pre-2009 cause-code continuity for tuberculosis and viral hepatitis**

| **Year** | **TB deaths, primary code** | **TB deaths, pre-2009 code** | **Old/new TB ratio (%)** | **Viral hepatitis deaths, HBV+HCV** | **Viral hepatitis deaths, pre-2009 code** | **New/old hepatitis ratio (%)** |
| --- | --- | --- | --- | --- | --- | --- |
| 2004 | 4314.00 | 4118.00 | 95.46 | 2955.00 | 2993.00 | 98.73 |
| 2005 | 4415.00 | 4214.00 | 95.45 | 2732.00 | 2778.00 | 98.34 |
| 2006 | 2758.00 | 2608.00 | 94.56 | 2510.00 | 2543.00 | 98.70 |
| 2007 | 2707.00 | 2534.00 | 93.61 | 2128.00 | 2158.00 | 98.61 |
| 2008 | 2523.00 | 2364.00 | 93.70 | 2318.00 | 2354.00 | 98.47 |

**Table S17. National annual burden of respiratory-parent and respiratory-subset categories, 2004-2021**

| **Year** | **Disease** | **Deaths** | **CMR**  **(/100 000)** | **ASMR (/100 000)** | **Share of all-cause deaths (%)** |
| --- | --- | --- | --- | --- | --- |
| 2004 | Respiratory infections | 8733 | 12.27 | 14.86 | 2.03 |
| 2005 | Respiratory infections | 8969 | 12.55 | 15.04 | 2.05 |
| 2006 | Respiratory infections | 6988 | 10.59 | 12.57 | 2.01 |
| 2007 | Respiratory infections | 8435 | 11.80 | 13.62 | 2.10 |
| 2008 | Respiratory infections | 8846 | 11.97 | 13.55 | 2.08 |
| 2009 | Respiratory infections | 9033 | 12.04 | 15.71 | 2.06 |
| 2010 | Respiratory infections | 9555 | 12.13 | 16.71 | 2.11 |
| 2011 | Respiratory infections | 9890 | 12.78 | 16.09 | 2.22 |
| 2012 | Respiratory infections | 10670 | 13.82 | 13.13 | 2.32 |
| 2013 | Respiratory infections | 26776 | 11.78 | 10.38 | 1.83 |
| 2014 | Respiratory infections | 30645 | 12.08 | 10.22 | 1.86 |
| 2015 | Respiratory infections | 29166 | 11.32 | 9.55 | 1.74 |
| 2016 | Respiratory infections | 30570 | 11.55 | 8.97 | 1.75 |
| 2017 | Respiratory infections | 31749 | 11.71 | 8.97 | 1.78 |
| 2018 | Respiratory infections | 33553 | 12.32 | 8.94 | 1.84 |
| 2019 | Respiratory infections | 34005 | 12.28 | 9.29 | 1.82 |
| 2020 | Respiratory infections | 24795 | 8.90 | 6.15 | 1.31 |
| 2021 | Respiratory infections | 24026 | 8.97 | 5.46 | 1.26 |
| 2004 | Lower respiratory infections | 7578 | 10.65 | 12.84 | 1.76 |
| 2005 | Lower respiratory infections | 7838 | 10.96 | 13.10 | 1.79 |
| 2006 | Lower respiratory infections | 6036 | 9.14 | 10.84 | 1.74 |
| 2007 | Lower respiratory infections | 7478 | 10.46 | 12.06 | 1.86 |
| 2008 | Lower respiratory infections | 8591 | 11.62 | 13.15 | 2.02 |
| 2009 | Lower respiratory infections | 8925 | 11.90 | 15.52 | 2.04 |
| 2010 | Lower respiratory infections | 9469 | 12.02 | 16.56 | 2.09 |
| 2011 | Lower respiratory infections | 9811 | 12.68 | 15.96 | 2.20 |
| 2012 | Lower respiratory infections | 10611 | 13.74 | 13.06 | 2.31 |
| 2013 | Lower respiratory infections | 26483 | 11.65 | 10.27 | 1.81 |
| 2014 | Lower respiratory infections | 30306 | 11.95 | 10.11 | 1.84 |
| 2015 | Lower respiratory infections | 28771 | 11.17 | 9.42 | 1.72 |
| 2016 | Lower respiratory infections | 30059 | 11.35 | 8.81 | 1.72 |
| 2017 | Lower respiratory infections | 31231 | 11.52 | 8.82 | 1.75 |
| 2018 | Lower respiratory infections | 33036 | 12.13 | 8.80 | 1.81 |
| 2019 | Lower respiratory infections | 33559 | 12.12 | 9.17 | 1.80 |
| 2020 | Lower respiratory infections | 24397 | 8.76 | 6.04 | 1.29 |
| 2021 | Lower respiratory infections | 23627 | 8.82 | 5.36 | 1.24 |
| 2004 | Upper respiratory infections | 1151 | 1.62 | 2.00 | 0.27 |
| 2005 | Upper respiratory infections | 1121 | 1.57 | 1.92 | 0.26 |
| 2006 | Upper respiratory infections | 948 | 1.44 | 1.73 | 0.27 |
| 2007 | Upper respiratory infections | 952 | 1.33 | 1.56 | 0.24 |
| 2008 | Upper respiratory infections | 246 | 0.33 | 0.39 | 0.06 |
| 2009 | Upper respiratory infections | 104 | 0.14 | 0.19 | 0.02 |
| 2010 | Upper respiratory infections | 83 | 0.11 | 0.15 | 0.02 |
| 2011 | Upper respiratory infections | 76 | 0.10 | 0.13 | 0.02 |
| 2012 | Upper respiratory infections | 55 | 0.07 | 0.07 | 0.01 |
| 2013 | Upper respiratory infections | 284 | 0.12 | 0.11 | 0.02 |
| 2014 | Upper respiratory infections | 331 | 0.13 | 0.11 | 0.02 |
| 2015 | Upper respiratory infections | 383 | 0.15 | 0.13 | 0.02 |
| 2016 | Upper respiratory infections | 495 | 0.19 | 0.15 | 0.03 |
| 2017 | Upper respiratory infections | 507 | 0.19 | 0.15 | 0.03 |
| 2018 | Upper respiratory infections | 506 | 0.19 | 0.14 | 0.03 |
| 2019 | Upper respiratory infections | 435 | 0.16 | 0.12 | 0.02 |
| 2020 | Upper respiratory infections | 389 | 0.14 | 0.10 | 0.02 |
| 2021 | Upper respiratory infections | 388 | 0.14 | 0.09 | 0.02 |

**Table S18. HIV/AIDS burden by broad age group at selected time points**

| **Year** | **Age group** | **Deaths** | **CMR (/100 000)** | **Share of all-cause deaths (%)** | **Share within combined infectious mortality (%)** |
| --- | --- | --- | --- | --- | --- |
| 2004 | 0-4 years | 3 | 0.08 | 0.03 | 0.14 |
| 2004 | 20-39 years | 71 | 0.28 | 0.27 | 5.51 |
| 2004 | 40-59 years | 71 | 0.41 | 0.09 | 2.26 |
| 2004 | 5-19 years | 4 | 0.02 | 0.06 | 0.87 |
| 2004 | 60-74 years | 7 | 0.11 | 0.01 | 0.15 |
| 2004 | 75+ years | 2 | 0.11 | 0.00 | 0.03 |
| 2013 | 0-4 years | 7 | 0.06 | 0.04 | 0.31 |
| 2013 | 20-39 years | 282 | 0.41 | 0.56 | 14.93 |
| 2013 | 40-59 years | 465 | 0.63 | 0.19 | 6.49 |
| 2013 | 5-19 years | 14 | 0.04 | 0.13 | 2.40 |
| 2013 | 60-74 years | 183 | 0.71 | 0.04 | 1.88 |
| 2013 | 75+ years | 78 | 0.87 | 0.01 | 0.34 |
| 2019 | 0-4 years | 2 | 0.01 | 0.02 | 0.16 |
| 2019 | 20-39 years | 337 | 0.44 | 0.78 | 18.62 |
| 2019 | 40-59 years | 664 | 0.76 | 0.26 | 8.71 |
| 2019 | 5-19 years | 7 | 0.02 | 0.07 | 1.34 |
| 2019 | 60-74 years | 377 | 0.93 | 0.07 | 3.14 |
| 2019 | 75+ years | 124 | 1.03 | 0.01 | 0.41 |
| 2021 | 0-4 years | 1 | 0.01 | 0.01 | 0.18 |
| 2021 | 20-39 years | 303 | 0.44 | 0.77 | 22.49 |
| 2021 | 40-59 years | 618 | 0.74 | 0.24 | 9.40 |
| 2021 | 5-19 years | 10 | 0.02 | 0.11 | 3.48 |
| 2021 | 60-74 years | 323 | 0.79 | 0.06 | 3.32 |
| 2021 | 75+ years | 151 | 1.07 | 0.01 | 0.67 |

*Note: CMR = crude mortality rate. Share within combined infectious mortality = percentage of combined infectious deaths attributable to HIV/AIDS within the same age group and year (denominator = all combined infectious deaths in that age group and year).* *For combined infectious mortality overall (all causes combined), the age-specific mortality ratio (75+ vs 0–4 years) increased from 6.4 in 2004 to 41.9 in 2021 (see main text, Figure 2), indicating a widening gap in infectious mortality risk between older and younger age groups beyond what is explained by population ageing alone.*

**Table S19. HIV/AIDS burden by detailed age band in 2004 and 2021**

| **Year** | **Detailed age band** | **Deaths** | **CMR (/100000)** | **Share of all-cause deaths (%)** |
| --- | --- | --- | --- | --- |
| 2004 | 0-4 years | 3 | 0.08 | 0.03 |
| 2004 | 5-9 years | 1 | 0.02 | 0.07 |
| 2004 | 10-14 years | 1 | 0.02 | 0.05 |
| 2004 | 15-19 years | 2 | 0.03 | 0.06 |
| 2004 | 20-24 years | 5 | 0.09 | 0.13 |
| 2004 | 25-29 years | 9 | 0.14 | 0.19 |
| 2004 | 30-34 years | 23 | 0.33 | 0.30 |
| 2004 | 35-39 years | 34 | 0.54 | 0.33 |
| 2004 | 40-44 years | 27 | 0.52 | 0.22 |
| 2004 | 45-49 years | 15 | 0.29 | 0.09 |
| 2004 | 50-54 years | 21 | 0.52 | 0.09 |
| 2004 | 55-59 years | 8 | 0.27 | 0.03 |
| 2004 | 60-64 years | 3 | 0.12 | 0.01 |
| 2004 | 65-69 years | 4 | 0.19 | 0.01 |
| 2004 | 70-74 years | 0 | 0.00 | 0.00 |
| 2004 | 75-79 years | 1 | 0.10 | 0.00 |
| 2004 | 80-84 years | 1 | 0.18 | 0.00 |
| 2004 | 85+ years | 0 | 0.00 | 0.00 |
| 2021 | 0-4 years | 1 | 0.01 | 0.01 |
| 2021 | 5-9 years | 0 | 0.00 | 0.00 |
| 2021 | 10-14 years | 2 | 0.01 | 0.06 |
| 2021 | 15-19 years | 8 | 0.06 | 0.20 |
| 2021 | 20-24 years | 29 | 0.21 | 0.66 |
| 2021 | 25-29 years | 58 | 0.38 | 0.89 |
| 2021 | 30-34 years | 91 | 0.42 | 0.74 |
| 2021 | 35-39 years | 125 | 0.71 | 0.79 |
| 2021 | 40-44 years | 154 | 0.85 | 0.65 |
| 2021 | 45-49 years | 167 | 0.76 | 0.37 |
| 2021 | 50-54 years | 152 | 0.65 | 0.20 |
| 2021 | 55-59 years | 145 | 0.73 | 0.13 |
| 2021 | 60-64 years | 96 | 0.64 | 0.08 |
| 2021 | 65-69 years | 132 | 0.85 | 0.07 |
| 2021 | 70-74 years | 95 | 0.92 | 0.04 |
| 2021 | 75-79 years | 72 | 1.10 | 0.03 |
| 2021 | 80-84 years | 58 | 1.27 | 0.02 |
| 2021 | 85+ years | 21 | 0.69 | 0.01 |

**Table S20. HIV/AIDS inequalities in 2021 by sex, residence, and region**

| **Dimension** | **Group** | **Deaths** | **CMR** | **ASMR** | **Share of all-cause deaths (%)** | **Rate ratio (95% CI)** | **p-value** |
| --- | --- | --- | --- | --- | --- | --- | --- |
| Sex | Female | 284 | 0.21 | 0.20 | 0.04 | 1.00 (ref) | — |
| Sex | Male | 1,122 | 0.82 | 0.78 | 0.10 | 3.90 (3.40–4.47) | <0.001 |
| Region | Eastern | 201 | 0.19 | 0.18 | 0.03 | 1.00 (ref) | — |
| Region | Central | 245 | 0.27 | 0.26 | 0.04 | 1.44 (1.21–1.71) | <0.001 |
| Region | Western | 960 | 1.35 | 1.26 | 0.19 | 7.00 (6.20–7.80) | <0.001 |
| Residence | Urban | 412 | 0.45 | 0.38 | 0.07 | 1.00 (ref) | — |
| Residence | Rural | 994 | 0.56 | 0.54 | 0.08 | 1.42 (1.27–1.59) | <0.001 |

**Note: CMR = crude mortality rate (per 100,000 population); ASMR = age-standardised mortality rate (per 100,000 population). Rate ratios for sex and region are based on ASMR values (male/female: 0.78/0.20 = 3.90; western/eastern: 1.26/0.18 = 7.00; central/eastern: 0.26/0.18 = 1.44). Rate ratio for residence is based on ASMR (0.54/0.38 = 1.42) with 95% confidence interval calculated using exact Poisson methods based on death counts (urban n=412, rural n=994).**

**Table S21. Interaction p values for heterogeneity in temporal trends**

| **Disease** | **Dimension** | **Interaction p, 2004-2021** | **Interaction p, 2013-2019** |
| --- | --- | --- | --- |
| Combined infectious mortality | Age group | 0.0000 | 0.0000 |
| Combined infectious mortality | Sex | 0.0629 | 0.5487 |
| Combined infectious mortality | Residence | 0.0003 | 0.6440 |
| Combined infectious mortality | Region | 0.0767 | 0.2042 |
| Respiratory infections | Age group | 0.0000 | 0.0016 |
| Respiratory infections | Sex | 0.0430 | 0.4652 |
| Respiratory infections | Residence | 0.0029 | 0.8769 |
| Respiratory infections | Region | 0.0640 | 0.1543 |
| Tuberculosis | Age group | 0.0000 | 0.0016 |
| Tuberculosis | Sex | 0.0007 | 0.2142 |
| Tuberculosis | Residence | 0.0022 | 0.5968 |
| Tuberculosis | Region | 0.1413 | 0.0846 |
| Viral hepatitis (HBV+HCV) | Age group | 0.0000 | 0.0816 |
| Viral hepatitis (HBV+HCV) | Sex | 0.5554 | 0.6636 |
| Viral hepatitis (HBV+HCV) | Residence | 0.5645 | 0.4897 |
| Viral hepatitis (HBV+HCV) | Region | 0.0227 | 0.2007 |
| HIV/AIDS | Age group | 0.0000 | 0.0758 |
| HIV/AIDS | Sex | 0.0000 | 0.0217 |
| HIV/AIDS | Residence | 0.1378 | 0.0793 |
| HIV/AIDS | Region | 0.0000 | 0.0566 |

**Table S22. Negligible national mortality from selected parasitic and tropical causes**

| **Cause** | **Peak year** | **Peak deaths** | **Peak CMR (/100 000)** | **2021 deaths** | **2021 CMR (/100 000)** |
| --- | --- | --- | --- | --- | --- |
| Malaria | 2005 | 10 | 0.01 | 11 | 0.00 |
| Tropical diseases | 2011 | 44 | 0.06 | 89 | 0.03 |
| Schistosomiasis | 2005 | 38 | 0.05 | 81 | 0.03 |
| Dengue | 2014 | 4 | 0.00 | 1 | 0.00 |
| Trichuriasis | 2009 | 0 | 0.00 | 0 | 0.00 |

**Table S23. National annual HIV/AIDS burden, 2004-2021**

| **Year** | **Deaths** | **CMR (/100 000)** | **ASMR (/100 000)** | **Share of all-cause deaths (%)** |
| --- | --- | --- | --- | --- |
| 2004.00 | 158.00 | 0.22 | 0.23 | 0.04 |
| 2005.00 | 186.00 | 0.26 | 0.28 | 0.04 |
| 2006.00 | 155.00 | 0.23 | 0.25 | 0.04 |
| 2007.00 | 232.00 | 0.33 | 0.33 | 0.06 |
| 2008.00 | 206.00 | 0.28 | 0.29 | 0.05 |
| 2009.00 | 218.00 | 0.29 | 0.29 | 0.05 |
| 2010.00 | 338.00 | 0.43 | 0.43 | 0.07 |
| 2011.00 | 262.00 | 0.34 | 0.33 | 0.06 |
| 2012.00 | 281.00 | 0.36 | 0.35 | 0.06 |
| 2013.00 | 1029.00 | 0.45 | 0.44 | 0.07 |
| 2014.00 | 1204.00 | 0.47 | 0.46 | 0.07 |
| 2015.00 | 1236.00 | 0.48 | 0.47 | 0.07 |
| 2016.00 | 1404.00 | 0.53 | 0.51 | 0.08 |
| 2017.00 | 1488.00 | 0.55 | 0.53 | 0.08 |
| 2018.00 | 1510.00 | 0.56 | 0.53 | 0.08 |
| 2019.00 | 1511.00 | 0.55 | 0.50 | 0.08 |
| 2020.00 | 1380.00 | 0.49 | 0.45 | 0.07 |
| 2021.00 | 1406.00 | 0.53 | 0.48 | 0.07 |

**Table S24. Sex-specific annual HIV/AIDS burden, 2004-2021**

| **Year** | **Sex** | **Deaths** | **CMR (/100 000)** | **ASMR (/100 000)** | **Share of all-cause deaths (%)** |
| --- | --- | --- | --- | --- | --- |
| 2004 | Female | 62 | 0.18 | 0.19 | 0.03 |
| 2004 | Male | 96 | 0.26 | 0.28 | 0.04 |
| 2005 | Female | 77 | 0.22 | 0.24 | 0.04 |
| 2005 | Male | 109 | 0.30 | 0.31 | 0.04 |
| 2006 | Female | 53 | 0.16 | 0.18 | 0.04 |
| 2006 | Male | 102 | 0.30 | 0.31 | 0.05 |
| 2007 | Female | 70 | 0.20 | 0.21 | 0.04 |
| 2007 | Male | 162 | 0.44 | 0.45 | 0.07 |
| 2008 | Female | 55 | 0.15 | 0.16 | 0.03 |
| 2008 | Male | 151 | 0.40 | 0.42 | 0.06 |
| 2009 | Female | 65 | 0.18 | 0.18 | 0.04 |
| 2009 | Male | 153 | 0.40 | 0.41 | 0.06 |
| 2010 | Female | 84 | 0.22 | 0.22 | 0.04 |
| 2010 | Male | 254 | 0.63 | 0.64 | 0.10 |
| 2011 | Female | 64 | 0.17 | 0.17 | 0.04 |
| 2011 | Male | 198 | 0.50 | 0.50 | 0.07 |
| 2012 | Female | 56 | 0.15 | 0.14 | 0.03 |
| 2012 | Male | 225 | 0.57 | 0.57 | 0.08 |
| 2013 | Female | 234 | 0.21 | 0.20 | 0.04 |
| 2013 | Male | 795 | 0.69 | 0.67 | 0.09 |
| 2014 | Female | 248 | 0.20 | 0.19 | 0.04 |
| 2014 | Male | 956 | 0.74 | 0.73 | 0.10 |
| 2015 | Female | 235 | 0.18 | 0.18 | 0.03 |
| 2015 | Male | 1001 | 0.77 | 0.75 | 0.10 |
| 2016 | Female | 283 | 0.22 | 0.21 | 0.04 |
| 2016 | Male | 1121 | 0.83 | 0.81 | 0.11 |
| 2017 | Female | 307 | 0.23 | 0.22 | 0.04 |
| 2017 | Male | 1181 | 0.86 | 0.83 | 0.11 |
| 2018 | Female | 280 | 0.21 | 0.20 | 0.04 |
| 2018 | Male | 1230 | 0.89 | 0.86 | 0.12 |
| 2019 | Female | 251 | 0.18 | 0.16 | 0.03 |
| 2019 | Male | 1260 | 0.90 | 0.84 | 0.12 |
| 2020 | Female | 252 | 0.18 | 0.16 | 0.03 |
| 2020 | Male | 1128 | 0.80 | 0.74 | 0.10 |
| 2021 | Female | 284 | 0.21 | 0.20 | 0.04 |
| 2021 | Male | 1122 | 0.82 | 0.78 | 0.10 |

**Table S25. Urban-rural annual HIV/AIDS burden, 2004-2021**

| **Year** | **Residence** | **Deaths** | **CMR (/100 000)** | **ASMR (/100 000)** | **Share of all-cause deaths (%)** |
| --- | --- | --- | --- | --- | --- |
| 2004 | Rural | 119 | 0.25 | 0.28 | 0.04 |
| 2004 | Urban | 39 | 0.16 | 0.16 | 0.03 |
| 2005 | Rural | 139 | 0.29 | 0.32 | 0.05 |
| 2005 | Urban | 47 | 0.20 | 0.19 | 0.03 |
| 2006 | Rural | 115 | 0.26 | 0.28 | 0.05 |
| 2006 | Urban | 40 | 0.19 | 0.18 | 0.04 |
| 2007 | Rural | 183 | 0.39 | 0.42 | 0.07 |
| 2007 | Urban | 49 | 0.20 | 0.20 | 0.04 |
| 2008 | Rural | 145 | 0.30 | 0.33 | 0.05 |
| 2008 | Urban | 61 | 0.24 | 0.23 | 0.04 |
| 2009 | Rural | 169 | 0.35 | 0.36 | 0.06 |
| 2009 | Urban | 49 | 0.18 | 0.18 | 0.03 |
| 2010 | Rural | 223 | 0.46 | 0.47 | 0.08 |
| 2010 | Urban | 115 | 0.39 | 0.37 | 0.07 |
| 2011 | Rural | 166 | 0.36 | 0.37 | 0.06 |
| 2011 | Urban | 96 | 0.31 | 0.28 | 0.06 |
| 2012 | Rural | 193 | 0.42 | 0.42 | 0.06 |
| 2012 | Urban | 88 | 0.29 | 0.27 | 0.06 |
| 2013 | Rural | 708 | 0.45 | 0.44 | 0.07 |
| 2013 | Urban | 321 | 0.46 | 0.42 | 0.07 |
| 2014 | Rural | 808 | 0.47 | 0.47 | 0.07 |
| 2014 | Urban | 396 | 0.49 | 0.46 | 0.08 |
| 2015 | Rural | 752 | 0.43 | 0.43 | 0.07 |
| 2015 | Urban | 484 | 0.58 | 0.55 | 0.09 |
| 2016 | Rural | 902 | 0.51 | 0.51 | 0.07 |
| 2016 | Urban | 502 | 0.57 | 0.53 | 0.09 |
| 2017 | Rural | 1037 | 0.58 | 0.57 | 0.09 |
| 2017 | Urban | 451 | 0.49 | 0.45 | 0.08 |
| 2018 | Rural | 1050 | 0.59 | 0.57 | 0.09 |
| 2018 | Urban | 460 | 0.49 | 0.46 | 0.08 |
| 2019 | Rural | 1022 | 0.56 | 0.53 | 0.08 |
| 2019 | Urban | 489 | 0.52 | 0.45 | 0.08 |
| 2020 | Rural | 906 | 0.50 | 0.46 | 0.07 |
| 2020 | Urban | 474 | 0.48 | 0.42 | 0.08 |
| 2021 | Rural | 994 | 0.56 | 0.54 | 0.08 |
| 2021 | Urban | 412 | 0.45 | 0.38 | 0.07 |

**Table S26. Region-specific annual HIV/AIDS burden, 2004-2021**

| **Year** | **Region** | **Deaths** | **CMR (/100 000)** | **ASMR (/100 000)** | **Share of all-cause deaths (%)** |
| --- | --- | --- | --- | --- | --- |
| 2004 | Eastern | 5 | 0.02 | 0.02 | 0.00 |
| 2004 | Central | 137 | 0.55 | 0.59 | 0.09 |
| 2004 | Western | 16 | 0.08 | 0.08 | 0.01 |
| 2005 | Eastern | 11 | 0.04 | 0.04 | 0.01 |
| 2005 | Central | 149 | 0.60 | 0.66 | 0.10 |
| 2005 | Western | 26 | 0.13 | 0.12 | 0.02 |
| 2006 | Eastern | 17 | 0.07 | 0.07 | 0.01 |
| 2006 | Central | 91 | 0.39 | 0.43 | 0.07 |
| 2006 | Western | 47 | 0.27 | 0.27 | 0.06 |
| 2007 | Eastern | 10 | 0.04 | 0.04 | 0.01 |
| 2007 | Central | 141 | 0.56 | 0.60 | 0.10 |
| 2007 | Western | 81 | 0.41 | 0.42 | 0.08 |
| 2008 | Eastern | 28 | 0.10 | 0.10 | 0.02 |
| 2008 | Central | 107 | 0.42 | 0.45 | 0.07 |
| 2008 | Western | 71 | 0.35 | 0.37 | 0.06 |
| 2009 | Eastern | 23 | 0.08 | 0.08 | 0.01 |
| 2009 | Central | 115 | 0.44 | 0.45 | 0.08 |
| 2009 | Western | 80 | 0.39 | 0.41 | 0.07 |
| 2010 | Eastern | 32 | 0.10 | 0.10 | 0.02 |
| 2010 | Central | 121 | 0.45 | 0.45 | 0.08 |
| 2010 | Western | 185 | 0.87 | 0.89 | 0.15 |
| 2011 | Eastern | 39 | 0.13 | 0.12 | 0.02 |
| 2011 | Central | 77 | 0.28 | 0.28 | 0.05 |
| 2011 | Western | 146 | 0.72 | 0.74 | 0.12 |
| 2012 | Eastern | 31 | 0.10 | 0.10 | 0.02 |
| 2012 | Central | 67 | 0.25 | 0.24 | 0.04 |
| 2012 | Western | 183 | 0.91 | 0.93 | 0.15 |
| 2013 | Eastern | 182 | 0.21 | 0.20 | 0.03 |
| 2013 | Central | 252 | 0.31 | 0.30 | 0.05 |
| 2013 | Western | 595 | 1.04 | 1.04 | 0.16 |
| 2014 | Eastern | 212 | 0.21 | 0.21 | 0.03 |
| 2014 | Central | 249 | 0.28 | 0.27 | 0.04 |
| 2014 | Western | 743 | 1.16 | 1.15 | 0.18 |
| 2015 | Eastern | 189 | 0.19 | 0.18 | 0.03 |
| 2015 | Central | 250 | 0.28 | 0.27 | 0.04 |
| 2015 | Western | 797 | 1.15 | 1.14 | 0.18 |
| 2016 | Eastern | 199 | 0.20 | 0.18 | 0.03 |
| 2016 | Central | 268 | 0.29 | 0.27 | 0.04 |
| 2016 | Western | 937 | 1.35 | 1.34 | 0.21 |
| 2017 | Eastern | 222 | 0.21 | 0.19 | 0.03 |
| 2017 | Central | 238 | 0.25 | 0.24 | 0.04 |
| 2017 | Western | 1028 | 1.48 | 1.47 | 0.23 |
| 2018 | Eastern | 253 | 0.24 | 0.22 | 0.03 |
| 2018 | Central | 279 | 0.29 | 0.28 | 0.04 |
| 2018 | Western | 978 | 1.39 | 1.37 | 0.21 |
| 2019 | Eastern | 258 | 0.24 | 0.21 | 0.04 |
| 2019 | Central | 275 | 0.29 | 0.27 | 0.04 |
| 2019 | Western | 978 | 1.34 | 1.27 | 0.20 |
| 2020 | Eastern | 230 | 0.21 | 0.19 | 0.03 |
| 2020 | Central | 288 | 0.30 | 0.28 | 0.04 |
| 2020 | Western | 862 | 1.17 | 1.07 | 0.17 |
| 2021 | Eastern | 201 | 0.19 | 0.18 | 0.03 |
| 2021 | Central | 245 | 0.27 | 0.26 | 0.04 |
| 2021 | Western | 960 | 1.35 | 1.26 | 0.19 |

**Table S27. Supplementary historical-context decomposition of the change in the proportion of combined infectious deaths among adults aged ≥75 years, China, 2004–2021**

| **Component** | **Contribution** |
| --- | --- |
| Total observed change (2004: 37.67% → 2021: 55.04%) | +17.37 percentage points |
| Population ageing effect | +9.92 pp (57.1%) |
| Mortality risk effect | +7.45 pp (42.9%) |

**Note: pp = percentage points. Although this decomposition spans the 2013 surveillance expansion and should not be used for direct trend inference, its directionality (population ageing and mortality risk changes both contribute) is consistent with the cross-sectional age-structure analysis in Table 3 of the main manuscript, supporting the conclusion that both factors drive the ageing of infectious mortality.*

**Table S28. Sensitivity analysis: age-standardised mortality rates for combined infectious mortality using alternative standard populations, China, 2013 and 2019**

| **Year** | **ASMR (2010 China Standard)** | **ASMR (WHO Standard)** |
| --- | --- | --- |
| 2013 | 17.60 | 2.71 |
| 2019 | 15.04 | 2.19 |

*Note: WHO standard population weights (2000–2025) were used as an alternative to the 2010 Chinese census standard population. Both standards show a consistent decline from 2013 to 2019.*

# Supplementary figures

**Figure S1. National crude mortality rates, China, 2004–2021**

*Dashed vertical lines indicate the 2013 surveillance system expansion and the 2020 pandemic onset.*

**Figure S2. National age-standardised mortality rates, China, 2004-2021**

**Figure S3. Shares of all-cause deaths, China, 2004-2021**

**Figure S4. Combined infectious mortality by broad age group, China, 2004–2021**

*Dashed vertical line indicates the 2013 surveillance system expansion (from 161 to 605 points). Data before and after 2013 are not directly comparable in level; trend comparisons should focus on periods within each surveillance era*

**Figure S5. Respiratory infections by broad age group, China, 2004–2021**

*Dashed vertical line indicates the 2013 surveillance system expansion (from 161 to 605 points). Data before and after 2013 are not directly comparable in level; trend comparisons should focus on periods within each surveillance era.*

**Figure S6. Tuberculosis, viral hepatitis, and HIV/AIDS by broad age group, China, 2004–2021**

*Dashed vertical line indicates the 2013 surveillance system expansion (from 161 to 605 points). Data before and after 2013 are not directly comparable in level; trend comparisons should focus on periods within each surveillance era.*

**Figure S7. Age redistribution of combined infectious deaths, China, 2004–2021**

*Dashed vertical line indicates the 2013 surveillance system expansion (from 161 to 605 points). Data before and after 2013 are not directly comparable in level; trend comparisons should focus on periods within each surveillance era.*

**Figure S8. Cause composition within combined infectious mortality by broad age group, China, 2004 and 2021**

**Figure S9. Sex-specific age-standardised mortality rates, China, 2021**

**Figure S10. Urban-rural age-standardised mortality rates, China, 2021**

**Figure S11. Region-specific age-standardised mortality rates, China, 2021**

**Figure S12. HIV/AIDS mortality by detailed age band, China, 2004 and 2021**

*The age distribution shifted towards middle-aged and older adults, especially between ages 40 and 74 years.*

**Figure S13. HIV/AIDS mortality by broad age group, China, 2004–2021**

**Figure S14.Respiratory sensitivity analysis: parent, lower, and upper respiratory categories, China, 2004–2021**

*Lower respiratory infections reproduced the parent-category pattern, whereas upper respiratory infections remained rare.*

**Figure S15. Inequality ratios over time, China, 2004–2021**

# Appendix references

1. Liu SW, Wu XL, Lopez AD, Wang LJ, Cai Y, Page A, et al. An integrated national mortality surveillance system for death registration and mortality surveillance, China. Bull World Health Organ 2016; 94: 46-57.

2. von Elm E, Altman DG, Egger M, Pocock SJ, Gøtzsche PC, Vandenbroucke JP, for the STROBE Initiative. The Strengthening the Reporting of Observational Studies in Epidemiology (STROBE) statement: guidelines for reporting observational studies. Lancet 2007; 370: 1453-57.

3. World Health Organization. Global hepatitis report 2024. Geneva: World Health Organization; 2024.

4. World Health Organization. Global tuberculosis report 2024. Geneva: World Health Organization; 2024.

5. Gao D, Zou Z, Dong J, Zhang Z, Yang D, Yang M. Age-period-cohort analysis of HIV mortality in China: data from the Global Burden of Disease Study 2016. Sci Rep 2020; 10: 7065.

6. Ren N, Li B, Zheng M, Dzakah EE, Zhao P, Liu S, et al. Patterns of HIV or AIDS mortality among older people from 1990 to 2019 in China: age-period-cohort analysis. JMIR Public Health Surveill 2022; 8: e35785.

7. Tang HL, Jin YC, Lyu F. HIV/AIDS epidemic in older adults and prevention and control challenges in China. Chinese Journal of Epidemiology 2023; 44: 1669–72 (in Chinese). doi:10.3760/cma.j.cn112338-20230629-00402.

8. Jin YC, Tang HL, Qin QQ, Cai C, Chen FF, Lyu F. Epidemiological characteristics and changing trends of reported people aged 60 years and older with HIV infection in China, 2015–2022. Chinese Journal of Epidemiology 2023; 44: 1673–78 (in Chinese). doi:10.3760/cma.j.cn112338-20230314-00146.

9. National Center for AIDS/STD Control and Prevention, Chinese Center for Disease Control and Prevention. Annual report of comprehensive prevention and treatment information for AIDS, sexually transmitted diseases, and hepatitis C. Beijing: National Center for AIDS/STD Control and Prevention, Chinese Center for Disease Control and Prevention; 2022 (in Chinese).

10. Chinese Center for Disease Control and Prevention. Technical guidelines for tuberculosis prevention and control in China, 2021 edition. Beijing: People's Medical Publishing House; 2021 (in Chinese).

11. Chen C, Liu G, Zeng G. Influenza disease burden in China: current status, challenges, and response strategies. Chinese Journal of Public Health 2022; 38: 1494–98 (in Chinese). doi:10.11847/zgggws1140026.

12. National Health Commission of the People's Republic of China. Screening and management of hepatitis C, WS/T 453-2014. Beijing: National Health Commission of the People's Republic of China; 2014 (in Chinese).
